# circPTPN12 promotes the progression and sunitinib resistance of renal cancer via hnRNPM/IL-6/STAT3 pathway

**DOI:** 10.1038/s41419-023-05717-z

**Published:** 2023-03-31

**Authors:** Yi Shou, Changjie Yue, Qi Wang, Jingchong Liu, Jiaju Xu, Qi Miao, Di Liu, Hongmei Yang, Yuenan Liu, Xiaoping Zhang

**Affiliations:** 1 Department of Urology, Union Hospital, Tongji Medical College, Huazhong University of Science and Technology, Wuhan, 430022 China; 2 Institute of Urologic Surgery, Tongji Medical College, Huazhong University of Science and Technology, Wuhan, 430022 China; 3 Department of Urology, Sir Run Run Shaw Hospital, Zhejiang University School of Medicine, Hangzhou, 310016 China; 4 Department of Pathogenic Biology, School of Basic Medicine, Huazhong University of Science and Technology, Wuhan, 430030 China

**Keywords:** Renal cell carcinoma, Prognostic markers

## Abstract

Renal cell carcinoma (RCC) is characterized by the difficulties in early diagnosis and the propensity to metastases. For advanced RCC, sunitinib targeted therapy is the clinically recommended first-line drug and the major challenge of sunitinib treatment is adaptive resistance. Therefore, it is imperative to research the mechanisms underlying sunitinib resistance. In this study, we discovered that circPTPN12 was highly expressed in RCC tissues and was associated with poorer clinical outcomes. circPTPN12 could promote the proliferation, migration, invasion, and sunitinib resistance of RCC cells. Mechanistically, circPTPN12 was found to form a complex with hnRNPM, which was involved in the regulation of mRNA processing. The combination with circPTPN12 enhanced the ability of hnRNPM to maintain the stability of IL-6 mRNA and further activated the STAT3 signaling pathway. The study revealed that circPTPN12/hnRNPM/IL-6/STAT3 axis promoted RCC progression and sunitinib resistance, which might be a promising therapeutic target for relieving sunitinib resistance in RCC.

## Introduction

Renal cell carcinoma (RCC) is the tumor that originated from renal tubular epithelial cells, which is one of the most common malignancies in the urinary system [1, 2]. The most recent research showed that the number of new renal cancer cases in the United States reached 76,080 in 2021, while the number of deaths caused by renal cancer reached 13,780 [3]. Due to the mild symptoms of RCC, nearly 30% of patients have distant metastases at the time of initial pathological diagnosis [4]. For patients who have lost the opportunity to have surgery for various reasons, sunitinib, one of the tyrosine kinase inhibitors (TKI), is currently the most commonly used first-line drug in clinical practice [5, 6]. Despite the fact that sunitinib can double the progression-free survival time of those patients, most of them eventually develop sunitinib resistance [7, 8]. Therefore, the identification of ideal early diagnostic markers for RCC and the thoroughly study of the unique mechanisms underlying adaptive sunitinib resistance are of utmost significance.

Circular RNAs (circRNAs) are covalently closed, endogenous biomolecules that are produced by backsplicing [9]. CircRNAs can contribute in a variety of biological processes in tumor cells, including as carcinogenesis, metastasis, immunological evasion, and drug resistance [10–13]. Numerous studies have documented the function of circRNAs in controlling tumor-targeted therapy tolerance [14–16]. In the field of renal cancer, relevant studies are still rare. The regulation of sunitinib resistance by circRNA in RCC has only been described in two studies [17, 18]. hsa_circ_0003764 (circPTPN12) is derived from the exons 5, 6, 7, and 8 regions within the PTPN12 locus. No studies concerning circPTPN12 have been reported in oncology research. Only two research in other disciplines have examined the capacity of circPTPN12 to encourage fibroblast proliferation in keloid and endometrial fibrosis [19, 20].

Multiple classical signaling pathways are involved in the development of sunitinib resistance in RCC including PI3K/AKT pathway, JAK/STAT pathway, and MAPK/ERK pathway [21–23]. When STAT3 is phosphorylated in the cytoplasm, it rapidly forms dimers and gathers in the nucleus to perform transcriptional functions, which has an impact on subsequent signaling molecules including PARP, c-MYC, and caspases-3 that promotes drug resistance [24–26].

In this study, circPTPN12 was found to be an upregulated circRNA in RCC that predicted poorer outcomes of RCC. circPTPN12 directly bound with hnRNPM and enhanced its ability to stabilize IL-6 mRNA, which led to the activation of STAT3 pathway and accelerated the progression and sunitinib resistance of RCC.

## Materials and methods

### RCC tissue samples

A total of 60 pairs of RCC tissues and corresponding normal tissues were collected from RCC patients undergoing radical nephrectomy or partial nephrectomy in the Department of Urology, Union Hospital, Tongji Medical College (Wuhan, China). Normal kidney tissue specimens were obtained from ≥2 cm from the edge of the resected kidney cancer tissue. These samples were used for subsequent whole transcriptome sequencing and qRT-PCR. All specimens were stored in an ultra-low temperature refrigerator at −80 °C immediately after a brief wash and information registration. All patients signed informed consent and agreed to participate in post-operative follow-up, and the research was approved by the Ethics Committee of Huazhong University of Science and Technology. All patients were followed up postoperatively by telephone as planned, by which overall survival time (OS) for these patients used for plotting survival curve was determined based on postoperative telephone follow-up on the last day of every month until the visit was lost or 5 years have elapsed.

### Cell culture

Human renal tubular epithelial cell line HK-2 and human RCC cell lines (786-O, ACHN, A-498, CAKI-1, and OSRC-2) were obtained from the American Type Culture Collection. All cells were cultured in high sugar DMEM medium containing 10% fetal bovine serum with 1% penicillin under 37 °C and 5% CO_2_. Sunitinib-resistant cell lines (786-O-R and ACHN-R) were induced by gradually raising the sunitinib concentration to 10 μM.

### Transient transfection and lentivirus transfection

Small interfering RNA (siRNA) targeting circPTPN12 (si-circPTPN12) and hnRNPM (si-hnRNPM) and the corresponding negative control siRNA (si-NC) were designed and provided by RiboBio (RibioBio, Guangzhou, China) and QijingBio (QijingBio, Wuhan, China) respectively. The truncated plasmids of hnRNPM were obtained from genecreate (Genecreate, Wuhan, China). The lentivirus used to overexpress circPTPN12 and a negative control lentivirus were purchased from Genechem (Genechem, Shanghai, China). For transient transfection, Lipofectamine 3000 reagent (Invitrogen, CA, USA) was used according to the instructions. Cells were harvested for subsequent experiments after 48 h of transfection. For lentivirus transfection, ZsGreen1 expression was assessed under a fluorescent microscope 3 days after transfection, and 5 μg/ml puromycin was used to screen the cells that were transfected. The information of the si-RNA, plasmids, and lentivirus were shown in Supplementary Table 1.

### Cell proliferation assays

1000 cells were seeded in the 96-well plates after 48 h of transfection. CCK-8 assay (CCK8; MCE, NJ, USA) was performed at the next 24, 48, 72, and 96 h. After 2 h of 10% CCK-8 incubation, the optical density values were measured by a spectrophotometer at 450 nm. EdU Cell Proliferation Assay Kit (Beyotime Biotechnology, Shanghai, China) was used to get a better visual of cell proliferation according to the manufacturer’s instructions. And the results were assessed under a fluorescent microscope.

### Cell migration and invasion assays

1 × 10^4^ cells were added into the upper chambers in serum-free medium for migration assay and 2 × 10^4^ cells were employed for invasion assay. After 24 h of incubation, the cells were fixed with 100% methanol and dyed with 0.05% crystal violet. Three randomly chosen fields were selected to count the cells across the membrane under ×100 and ×400 magnification.

### Quantitative reverse transcription PCR

Total RNA was extracted from tissues or cells using TRIzol® reagent (Thermo Fisher Scientific, MA, USA). The concentration and purity of the RNA were evaluated by a NanoDrop 2000 spectrophotometer (Thermo Fisher Scientific). Superscript II Reverse Transcription Kit (Takara Bio, Beijing, China) was used to reverse transcribe the RNA into cDNA according to the manufacturer’s protocol. The primers used in the study were synthesized by TSINGKE (TSINGKE, Beijing, China) and the sequences of these primers were shown in Supplementary Table 2.

### Western blot assays

Total protein was extracted from tissues or cells using RIPA lysis buffer (Servicbio.Inc., Wuhan, China) with 1% protease inhibitor Cocktail (MCE). After that, the protein concentration was adjusted to the same level using a BCA protein assay kit (Beyotime Biotechnology). After the electrophoresis and transmembrane, PVDF membranes (Millipore, Bedford, USA) were first incubated in 5% BSA (Servicbio.Inc.) for 2 h, then transferred to primary antibodies overnight at 4 °C after being rinsed with PBS. Next, the membranes were incubated in corresponding secondary antibodies for 1 h. Finally, the protein bands were detected using ChemiDoc XRS+ (Bio‑Rad Laboratories, CA, USA) with ECL chemiluminescent substrate (Biosharp, Wuhan, China). Information of the antibodies were shown in Supplementary Table 3.

### Actinomycin D treatment and RNA stability assays

The cells were treated with 10 μg/ml Actinomycin D (Selleck, TX, USA) for 0, 3, 6, 9, and 12 h when reached the fusion of 70%. Total RNA was extracted after Actinomycin D treatment. The RNA was analyzed by qRT-PCR with U6 as internal reference.

### Cellular fractionation assay of RNA

The cellular fractionation assay was conducted using Cytoplasmic & Nuclear RNA Purification Kit (Biotek, VT, USA). GAPDH was used as cytoplasmic internal reference and U6 was used as nuclear internal reference. The RNA was analyzed by qRT-PCR.

### RNA fluorescence in situ hybridization (FISH)

Fluorescent in Situ Hybridization kit (RiboBio) was used, in which circPTPN12, as well as U6 and 18S probes were labeled with Cy3 when cell fusion reached 70% in 24-well plates. Hybridization was performed in 786-O cells according to the manufacturer’s protocol. 0.1% DAPI was used to label the nucleus at last. Detection of individual channel fluorescence signals using a confocal laser scanning microscope (Carl Zeiss LSM 780, Baden-Württemberg, Germany).

### Immunofluorescence (IF)

786-O and ACHN cells were fixed with 100% methanol when the fusion reached 70% in 24-well plates. Then 0.1% Triton X-100 (Servicbio.Inc.) was used to increase the permeability of antibodies to cell membranes for 15 min. After that, the cells were closed with 5% BSA for 30 min at 37 °C and washed with PBS three times. Then the cells were incubated with hnRNPM antibody at 4 °C overnight. After washing the antibody with PBS, the cells were incubated with Fluorescent secondary antibody in light-proof conditions for 2 h. Finally, DAPI was used to label the nucleus. The images were acquired with a confocal laser scanning microscope (Carl Zeiss LSM 780).

### RNA pulldown assays

Biotin-labeled circPTPN12 (sense) and negative control (anti-sense) probes were designed by TSINGKE (TSINGKE). About 10^7^ cells were washed with pre-cooled PBS buffer and irradiated for 15 min under an ultraviolet crosslinker. Then the cells were incubated in lysis buffer (20Mm Tris-HCl, 150 mM NaCl, 1 mM EDTA, and 0.5% NP-40 with DTT, PMSF, Cocktail, and RNase inhibitor added before use). The probes were incubated in the cell lysates with rotation for 1 h at 4 °C. After that, the mixture was incubated with Streptavidin Magnetic Beads (MCE) for 2 h at 4 °C. Then the beads were washed with buffer A (25 mM Tris-HCl, 150 mM KCl, 5 mM EDTA, and 0.5% NP-40). The beads were heated for 10 min with 2 × protein loading buffer (Servicebio) at 95 °C. The sequences of biotin probes were list in Supplementary Table 4.

### Silver staining and mass spectrometry analysis

Protein silver stain plus kit (Coolaber, Beijing, China) was used to stain the protein gel as the manufacturer’s protocol described. The mass spectrometry analysis was accomplished by Novogene (Novogene, Beijing, China). Finally, Proteome Discoverer software (Thermo Fisher Scientific) was used to identify the proteins.

### RNA immunoprecipitation (RIP)

Magna RIP RNA-Binding Protein Immunoprecipitation Kit (Millipore) was applied in the RIP assay. About 10^7^ 786-O cells were collected and incubated in RIP lysis buffer at −80 °C. Then 5 μg antibodies were used to incubate with the cell lysates in Protein A/G Magnetic Beads (MCE) with rotation for 1 h. The magnetic beads were digested by proteinase K buffer in a shaker at 55 °C for 30 min. RNeasy MinElute Cleanup Kit (Qiagen, Hilden, Germany) was then applied to withdraw the RNA in the supernatant. The information of truncated plasmids was list in Supplementary Table 5.

### Tumor xenograft model

The BALB/c nude mice (4 weeks old, male) obtained from Beijing HFK Bio-Technology Co., Ltd. were chosen for tumor xenograft model. 2 × 10^6^ cells were injected subcutaneously into the axilla of the mice. The mice were intraperitoneally injected with stattic (3.75 mg/kg) or DMSO (MCE) and the size of the subcutaneous tumors was measured every 3 days. The mice were killed after 30 days. Afterward, the weight of tumors was measured and immunohistochemical staining assays were conducted.

### Immunohistochemistry assay

The tumor xenografts were successively fixed in 4% formalin at room temperature, dehydrated, and embedded in paraffin. The sections were incubated in p-STAT3 primary antibody overnight at 4 °C. After washing with PBS, the sections were incubated with corresponding secondary antibodies for 2 h. A NanoZoomer S360 (Hamamastu Corporation, Shizuoka, Japan) was used to scan the sections. Random fields were selected under ×100 and ×400 magnification.

### Bioinformatics analysis

The gene set enrichment analysis (GSEA) software (https://www.gsea-msigdb.org/gsea/index.jsp) and Kyoto Encyclopedia of Genes and Genomes databases (c2.all.v6.2.symbols.gmt) was used to find pathways enriched in the gene set on the basis of pathway Enrichment Score. The crystallographic data for static was downloaded from MCE website (https://www.medchemexpress.cn/Stattic.html).

### Statistical analysis

The results of the experiments were expressed as mean ± standard deviation (mean ± SD). Statistical analyses were performed by Graphpad 8.0 (GraphPad Software, Inc., CA, USA). Statistical differences between the two groups were compared using the student’s test. *P* < 0.05 indicated a statistical difference (*P < 0.05; **P < 0.01; ***P < 0.001; ****P < 0.0001). Three independent biological replicates were performed for each experiment. Heatmaps in the study were made using R 4.1.3.

## Results

### circPTPN12 is upregulated and associated with a worse prognosis in RCC

**Fig. 1:**
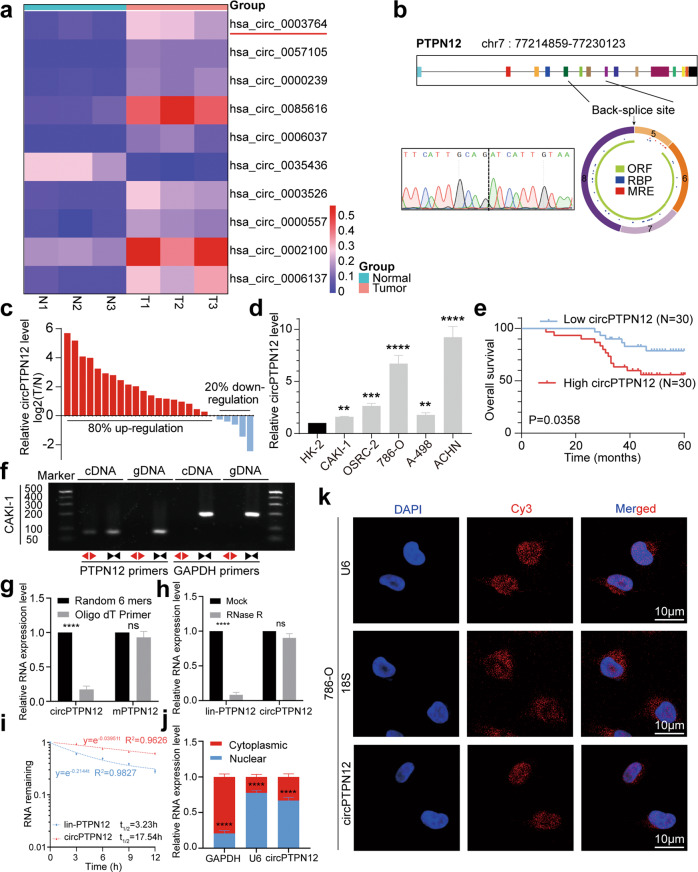
circPTPN12 is upregulated and associated with a worse prognosis in RCC. **a** The expression levels of ten differential expressed circRNAs with smallest *P* values in RCC. **b** Scheme illustrating the formation of circPTPN12, the back-splice site of which was verified by Sanger sequencing. **c** The relative expression levels of circPTPN12 in 20 pairs of RCC tissues and paired normal tissues. **d** The expression levels of circPTPN12 in normal cell line (HK-2) and RCC cell lines (786-O, ACHN, A-498, CAKI-1, and OSRC-2). **e** The Kaplan-Meier curves of circPTPN12 in RCC for overall survival time. **f** The existence of circPTPN12 was validated using divergent primers and convergent primers in cDNA. **g** circPTPN12 was conformed to lack poly-A tail using random 6 mers and oligo dT Primer. **h** The relative levels of circPTPN12 and PTPN12 were detected in the presence of RNase R. **i** The relative levels of circPTPN12 and PTPN12 were detected by qRT-PCR after actinomycin D treatment. **j** Identification of the proportion of circPTPN12 in the cytoplasm and nucleus. **k** The subcellular location of circPTPN12 was demonstrated by FISH assay. U6 and 18 S were used as positive controls for nucleus and cytoplasm respectively. Scale bars, 10 μm. ns: not significant, **P < 0.01, ***P < 0.001, ****P < 0.0001. The results were expressed with mean ± SD. All the experiments were replicated three times.

Three pairs of RCC tissues and the adjacent normal tissues were chosen for whole transcriptome RNA sequencing. The top 10 differential expressed circRNAs with the smallest P values were selected according to the screening criteria of |log2FC| ≥ 1.5, *P* < 0.05. Among these circRNAs, we found hsa_circ_0003764 was upregulated in RCC and had not been reported in the field of oncology research (Fig. 1a). hsa_circ_0003764 was derived from the exons 5, 6, 7, and 8 regions within the PTPN12 locus according to the CSCD database and the back-spliced site of which was validated by Sanger sequencing (Fig. 1b). circPTPN12 was significantly upregulated in more RCC tissues and RCC cell lines (Fig. 1c, d). Moreover, higher circPTPN12 levels indicated poorer clinical outcomes (Fig. 1e). DNA gel electrophoresis of RT-PCR products showed that circPTPN12 could be amplified with divergent primers in the cDNA but not gDNA (Fig. 1f). We reverse transcribed RNA with random 6 mers and oligo dT primers and performed qRT-PCR to calculate relative RNA levels. The result indicated that random 6 mers amplified circPTPN12 more efficiently than oligo dT primers compared to mPTPN12, which demonstrated that circPTPN12 lacked a poly-A tail structure (Fig. 1g). And circPTPN12 turned out to be more stable compared with its linear transcripts under Actinomycin D and RNase R treatment (Fig. 1h, i). qRT-PCR analysis of the nuclear/cytoplasmic fractionation and FISH assay indicated that circPTPN12 was mainly distributed in the nucleus of RCC cells (Fig. 1j, k).

### circPTPN12 promotes the proliferation, migration, and invasion of RCC cells in vitro

**Fig. 2:**
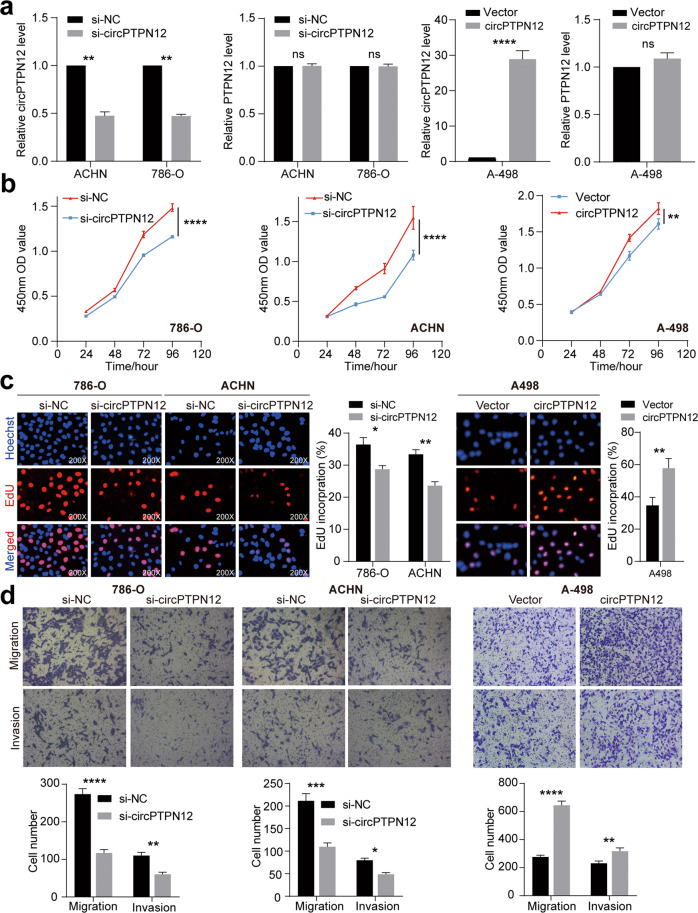
circPTPN12 promotes the proliferation, migration, and invasion of RCC cells in vitro. **a** The expression levels of circPTPN12 and PTPN12 after transfection. **b** The viability of RCC cells after knocking down and overexpressing circPTPN12 was detected by CCK-8 assay. **c** The proliferation ability of RCC cells after knocking down and overexpressing circPTPN12 was detected by EdU assay. **d** The migration and invasion abilities of RCC cells after transfection were detected by transwell assay. ns: not significant, *P < 0.05, **P < 0.01, ***P < 0.001, ****P < 0.0001. The results were expressed with mean ± SD. All the experiments were replicated three times.

To explore the biological function of circPTPN12 in RCC cells, siRNA and plasmids were used to knock down and overexpress circPTPN12. 786-O and ACHN cells with relatively high circPTPN12 expression were used to knock down circPTPN12, while A-498 cells with relatively low circPTPN12 expression were used to overexpress circPTPN12. qRT-PCR was applied to verify the effect of transient transfections (Fig. 2a). CCK-8 assays showed that the silencing of circPTPN12 significantly suppressed the proliferation ability of 786-O and ACHN cells, while the overexpressing of circPTPN12 facilitated the proliferative ability of A-498 cells (Fig. 2b). The results of EdU assays provided better visual of the effect of circPTPN12 on cell proliferation (Fig. 2c). Next, transwell assays were used to measure the migration and invasion ability of RCC cells. The results showed that circPTPN12 knockdown reduced the migration and invasion rates of 786-O and ACHN cells, while circPTPN12 overexpression promoted the above abilities of A-498 cells (Fig. 2d).

### circPTPN12 activates STAT3 signaling pathway in RCC cells

**Fig. 3:**
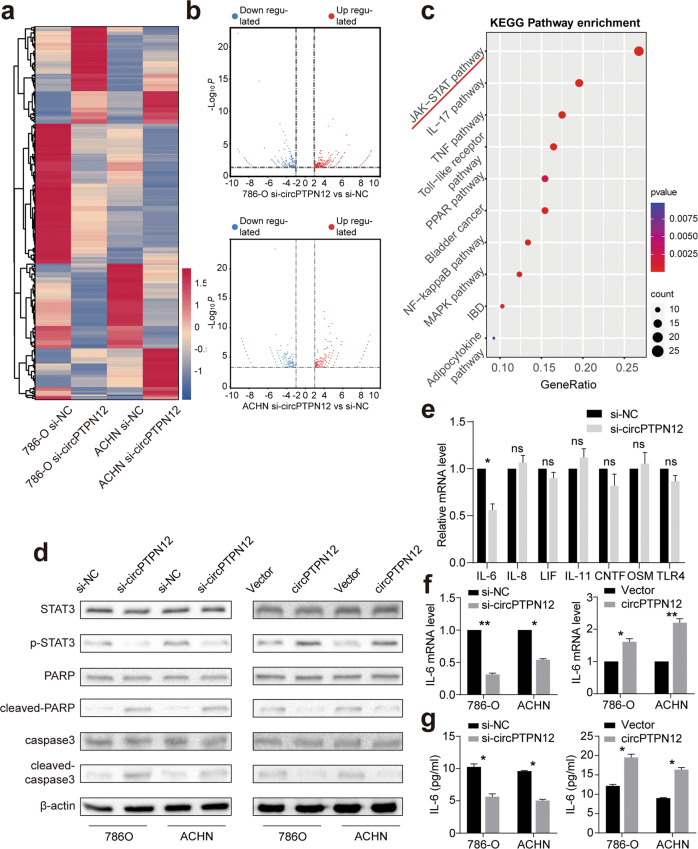
circPTPN12 activates STAT3 signaling pathway in RCC cells. **a** The heatmap of differential expressed genes in 786-O and ACHN cells after knocking down circPTPN12. **b** The volcano plot of differential expressed genes in 786-O and ACHN cells. **c** KEGG enrichment analysis of differential expressed genes. **d** The changes of STAT3 signaling pathway-related proteins after knocking down and overexpressing circPTPN12. **e** The changes of upstream molecules of STAT3 pathway after knocking down circPTPN12. **f** The changes of IL-6 mRNA levels after knocking down and overexpressing circPTPN12. **g** The changes of IL-6 secretion in supernatant after knocking down and overexpressing circPTPN12. ns: not significant, *P < 0.05, **P < 0.01. All the experiments were replicated three times.

We performed RNA sequencing (RNA-seq) using circPTPN12-knockdown and control cells to investigate the signaling pathways regulated by circPTPN12 in RCC. According to the test reports, we took an intersection of the differential genes from two cell lines and a total of 298 shared differential genes were obtained (Fig. 3a, b). KEGG enrichment analysis of these differential genes was further applied, among which the JAK-STAT pathway was the most significantly altered pathway with the smallest P value (Fig. 3c). We further verified that knockdown of circPTPN12 resulted in the reduction of phosphorylated STAT3 protein levels, as well as the increases of cleaved-PARP and cleaved-caspase3 levels. While the overexpression of circPTPN12 led to opposite changes in these proteins (Fig. 3d). Since circPTPN12 was mainly localized in the nucleus, whereas STAT3 phosphorylation occurred in the cytoplasm, we suggested circPTPN12 might activate the STAT3 pathway through the upstream molecules of this pathway instead of combining directly with STAT3. We selected IL6, IL-8, IL-11, LIF, CNTF, OSM, and TLR4, the reported upstream molecules of STAT3, and measured the mRNA levels of these molecules after knocking down circPTPN12 by qRT-PCR. The result suggested that only the level of IL-6 was reduced after circPTPN12 silencing (Fig. 3e). We further found a positive correlation between circPTPN12 levels and IL-6 secretion in the supernatant using ELISA experiments (Fig. 3f, g).

### circPTPN12 promotes sunitinib resistance by IL-6/STAT3 pathway in RCC cells

**Fig. 4:**
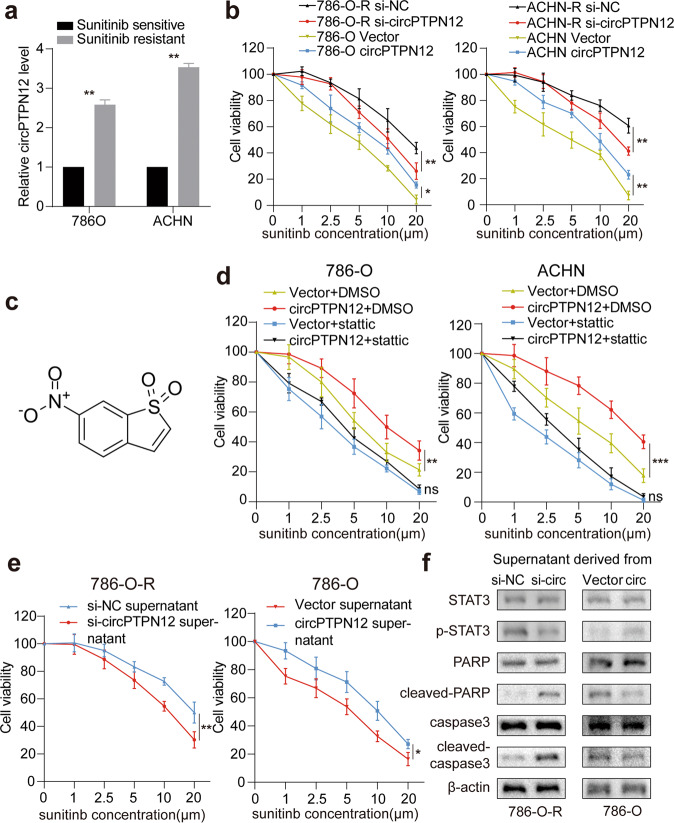
circPTPN12 promotes sunitinib resistance via IL-6/STAT3 pathway in RCC cells. **a** circPTPN12 was upregulated in sunitinib-resistant cells. **b** circPTPN12 promoted sunitinib resistance of RCC cells. **c** The molecular formula of stattic. **d** The sunitinib resistance of RCC cells caused by circPTPN12 could be reversed by stattic. **e** The changes of sunitinib sensitiveness of 786-O cells when incubated with supernatant derived from cells after knocking down or overexpressing circPTPN12. **f** The changes of STAT3 signaling pathway-related proteins when incubated with supernatant derived from cells after knocking down or overexpressing circPTPN12. ns: not significant, *P < 0.05, **P < 0.01, ***P < 0.001. The results were expressed with mean ± SD. All the experiments were replicated three times.

Since numerous studies showed that the STAT3 signaling pathway was involved in multiple drug resistance, it was natural to wonder whether circPTPN12 would affect sunitinib resistance in RCC cells [27–29]. We found that circPTPN12 was significantly upregulated in 786-O and ACHN sunitinib-resistant cells (Fig. 4a). We further selected sunitinib-resistant cells for knocking down circPTPN12 and parental cells for overexpressing circPTPN12, then tested its effects on sunitinib resistance. The results of the drug sensitivity curve indicated that circPTPN12 promoted the sunitinib resistance in RCC cells (Fig. 4b). To demonstrate that circPTPN12-induced sunitinib resistance was mediated by the STAT3 signaling pathway, stattic, a specific inhibitor of STAT3 phosphorylation, was used to apply rescue assays [30–32]. And the molecular formula of stattic was shown in Fig. 4c. The results showed that circPTPN12-induced sunitinib resistance could be reversed by stattic, which implied that circPTPN12 facilitated sunitinib resistance in RCC cells via the STAT3 pathway (Fig. 4d). We further verified whether circPTPN12 activated STAT3 pathway by increasing the IL-6 levels in cell supernatant. We discovered that the sunitinib resistance of 786-O-R cells was significantly reduced when cells were cultured with supernatant obtained from circPTPN12-knockdown cells, while the opposite changes were found when cells were incubated with supernatant from circPTPN12-overexpress cells (Fig. 4e). Finally, we examined the changes of STAT3 signaling pathway-related proteins in 786-O cells after supernatant incubation. The results showed a positive correlation between the level of IL-6 in the supernatant and the activation of the STAT3 pathway (Fig. 4f). These results demonstrated that circPTPN12 could promote sunitinib resistance through the IL-6/STAT3 pathway in RCC cells.

### circPTPN12 interacts with hnRNPM protein in RCC cells

**Fig. 5:**
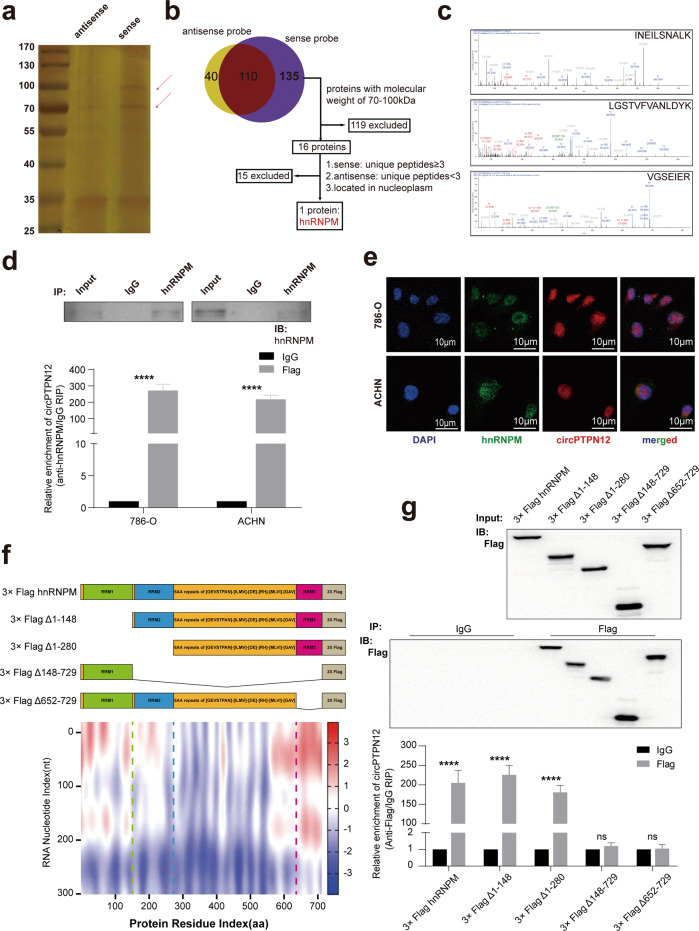
circPTPN12 interacts with hnRNPM protein in RCC cells. **a** Protein gel silver staining of circPTPN12 pull-down products. **b** The screening procedure for proteins that interact with circPTPN12. **c** Protein profiling demonstrated the possible peptides binding to circPTPN12 in the hnRNPM structure. **d** RIP experiments showed that hnRNPM gathered more circPTPN12 than the IgG group. **e** FISH experiments showed that both circPTPN12 and hnRNPM were predominantly localized in the nucleus. **f** Prediction of circPTPN12-hnRNPM interaction by catRAPID algorithm and the schematic of hnRNPM with RNA recognition motifs according to Uniprot database. hnRNPM truncations lacking the region 1–148 aa (3xFlag Δ1–148), 1–280 aa (3xFlag Δ1–280), 148–729 aa (3xFlag Δ148–729), or 652–729 aa (3xFlag Δ652–729). **g** The relative enrichment levels of endogenous circPTPN12 analyzed by truncated hnRNPM RIP assays. ns: not significant, *P < 0.05, ****P < 0.0001. The results were expressed with mean ± SD. All the experiments were replicated three times.

Since circPTPN12 was mainly distributed in the nucleus, where circRNAs exerted their subsequent effects mainly through binding proteins. RNA pulldown experiments and protein profiling analysis were used to further explore circPTPN12-binding proteins, which was important for the ensuing mechanistic studies. The ability of circPTPN12 to bind with AGO2 protein was the basis for ceRNA mechanistic studies, with which we could decide whether to conduct a ceRNA mechanistic study or a RBP mechanistic study [33–35]. We thus performed circPTPN12 RNA pull-down experiments to explore the proteins binding with circPTPN12 (Fig. 5a). Following the screening pipeline (Fig. 5b), a major differential protein band was confirmed to be hnRNPM by mass spectrometry analysis (Fig. 5c). circPTPN12 was validated to interact with hnRNPM through detecting the precipitates immunoprecipitated by anti-hnRNPM antibody (Fig. S1a) and RIP analysis (Fig. 5d). Besides, we did not find AGO2 in the protein profile results file, which implied that the ceRNA mechanism was not the major regulation mode of circPTPN12 in RCC (Supplementary Table 6). We further proved that circPTPN12 and hnRNPM had the same subcellular localization by FISH and IF assays (Fig. 5e). Moreover, catRAPID algorithm was used to predict the binding site and truncated plasmids were constructed according to the protein functional structural domains of hnRNPM displayed in UniProt database (Fig. 5f). The results of anti-Flag RIP showed that the RRM3 (RNA recognition motif 3, aa652–729) of hnRNPM was the key to the binding of hnRNPM and circPTPN12 (Fig. 5g).

### circPTPN12-hnRNPM complex improves the stability of IL-6 mRNA and maintains the activation of STAT3 pathway

Gene set enrichment analysis (GSEA) showed that hnRNPM was involved in the regulation of spliceosomes, the JAK-STAT signaling pathway, and RNA degradation (Fig. S1b, c). Next, we found that changing the expression of circPTPN12 or hnRNPM left the expression of the other unaffected, which suggested that the regulation of hnRNPM induced by circPTPN12 was not based on the expression level (Fig. S1d, e). We next investigate whether hnRNPM regulated the level of IL-6 mRNA by regulating its alternative splicing. IL-6 was reported to have three isoforms, among which IL-6 isoform 2 had been reported as an IL-6 inhibitor in RCC [36]. However, the level of all these three isoforms decreased when hnRNPM was knocked down and there was no increase in the expression of IL-6 isoform 2 as expected, suggesting that the change in expression levels of isoforms might be due to the changes in IL-6 mRNA level but not to hnRNPM-mediated alternative splicing (Fig. S1f).

**Fig. 6:**
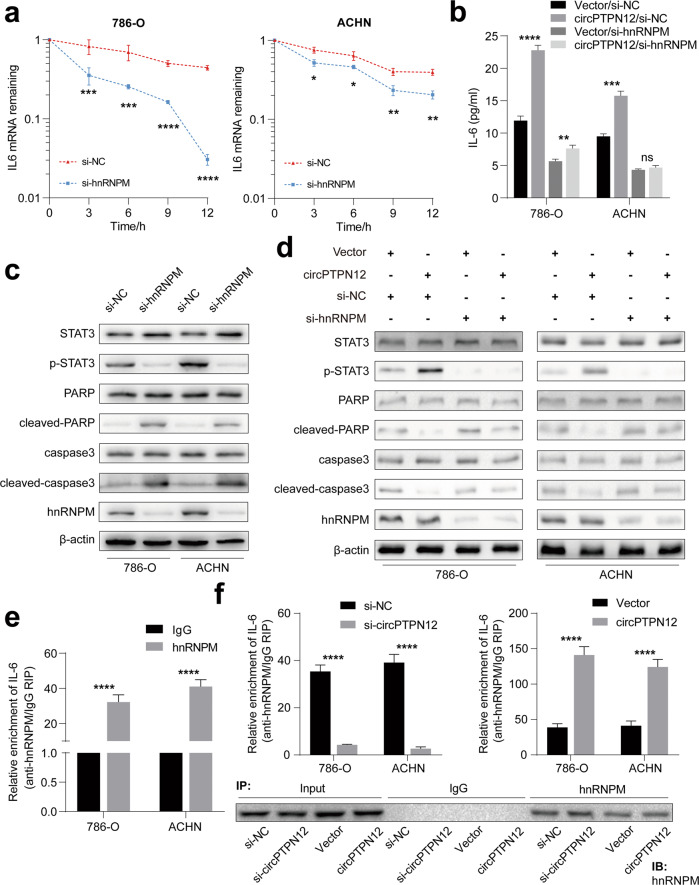
circPTPN12-hnRNPM complex improves the stability of IL-6 mRNA and maintains the activation of STAT3 pathway. **a** Actinomycin D assays showed that the stability of IL-6 mRNA decreased after hnRNPM silencing. **b** The rescue assays demonstrated that hnRNPM could reverse the effect of circPTPN12 on IL-6 secretion. **c** STAT3 pathway was suppressed after knocking down hnRNPM. **d** hnRNPM could reversed the effect of circPTPN12 on STAT3 signaling pathway. **e** hnRNPM enriched more IL-6 mRNA than IgG according to anti-hnRNPM RIP experiment. **f** Anti-hnRNPM RIP experiment showed that the level of IL-6 gathered by hnRNPM was regulated by circPTPN12 levels. ns: not significant, *P < 0.05, **P < 0.01, ***P < 0.001, ****P < 0.0001. The results were expressed with mean ± SD. All the experiments were replicated three times.

Recently, hnRNPM was reported to regulate the stability of target mRNA [37]. We speculated that hnRNPM might promote the accumulation of IL-6 by enhancing the stability of IL-6 mRNA. Actinomycin D assays demonstrated that the degradation rate of IL-6 mRNA was notably increased when hnRNPM was knocked down (Fig. 6a). Results of ELISA assays showed that IL-6 secretion in cell supernatants increased after overexpression of circPTPN12, whereas the secretion of IL-6 increased only slightly or even remained unchanged after hnRNPM inhibition (Fig. 6b). And the STAT3 pathway was inhibited after knockdown of hnRNPM (Fig. 6c). Moreover, the activation of STAT3 pathway caused by overexpression of circPTPN12 could also be reversed by hnRNPM silencing (Fig. 6d), which suggested that circPTPN12 activated IL-6/STAT3 pathway via hnRNPM. We further explored the mechanism by which hnRNPM improved the stability of IL-6 mRNA. We re-performed qRT-PCR to analyze the RNA products obtained from RIP experiments. The result indicated that hnRNPM could enrich more IL-6 mRNA than IgG (Fig. 6e). At last, we performed RIP experiments after knockdown and overexpression of circPTPN12. The results showed that the level of IL-6 enriched by hnRNPM was regulated by circPTPN12 levels (Fig. 6f). The above results suggested that circPTPN12 increased the binding level of hnRNPM and IL-6 mRNA, which might improve the ability of hnRNPM to stabilize the IL-6 mRNA.

### circPTPN12 promotes the progression and sunitinib resistance of RCC cells through hnRNPM

**Fig. 7:**
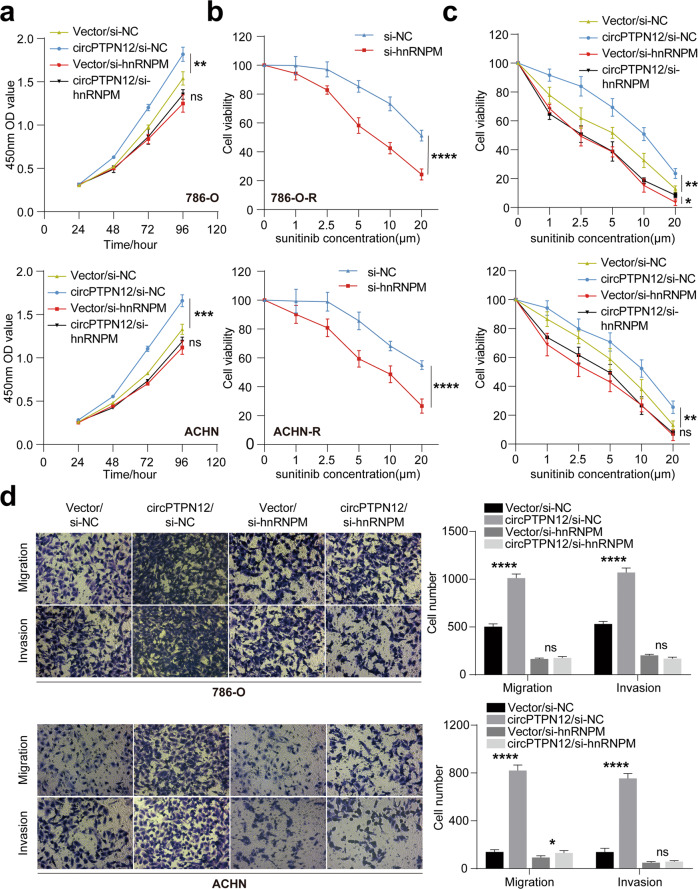
circPTPN12 promotes the progression and sunitinib resistance of RCC cells through hnRNPM. **a** CCK-8 assays showed that hnRNPM reversed the proliferative effects of circPTPN12. **b** Knocking down hnRNPM reduced the sunitinib resistance of 786-O-R and ACHN-R cells. **c** hnRNPM reversed the effect of circPTPN12 on sunitinib resistance. **d** Transwell assays indicated that hnRNPM reversed the effect of circPTPN12 on migration and invasion abilities. ns: not significant, *P < 0.05, **P < 0.01, ***P < 0.001, ****P < 0.0001. The results were expressed with mean ± SD. All the experiments were replicated three times.

We next investigated the effect of hnRNPM on the biological functions of RCC cells. We first examined the effect of hnRNPM on cell proliferation. The results of CCK-8 rescue assays demonstrated that hnRNPM could reverse the proliferative effect of circPTPN12 strongly (Fig. 7a). The drug sensitivity curve showed that the sunitinib resistance of RCC cells reduced significantly after knockdown of hnRNPM (Fig. 7b). And the sunitinib resistance caused by circPTPN12 could be reversed by hnRNPM silencing (Fig. 7c). Ultimately, transwell rescue assays showed that the abilities of migration and invasion of RCC cells were also reversed by hnRNPM silencing (Fig. 7d). The above results indicated that circPTPN12 promoted the progression and sunitinib resistance of RCC cells via hnRNPM.

### circPTPN12 accelerates tumor growth and sunitinib resistance in vivo

**Fig. 8:**
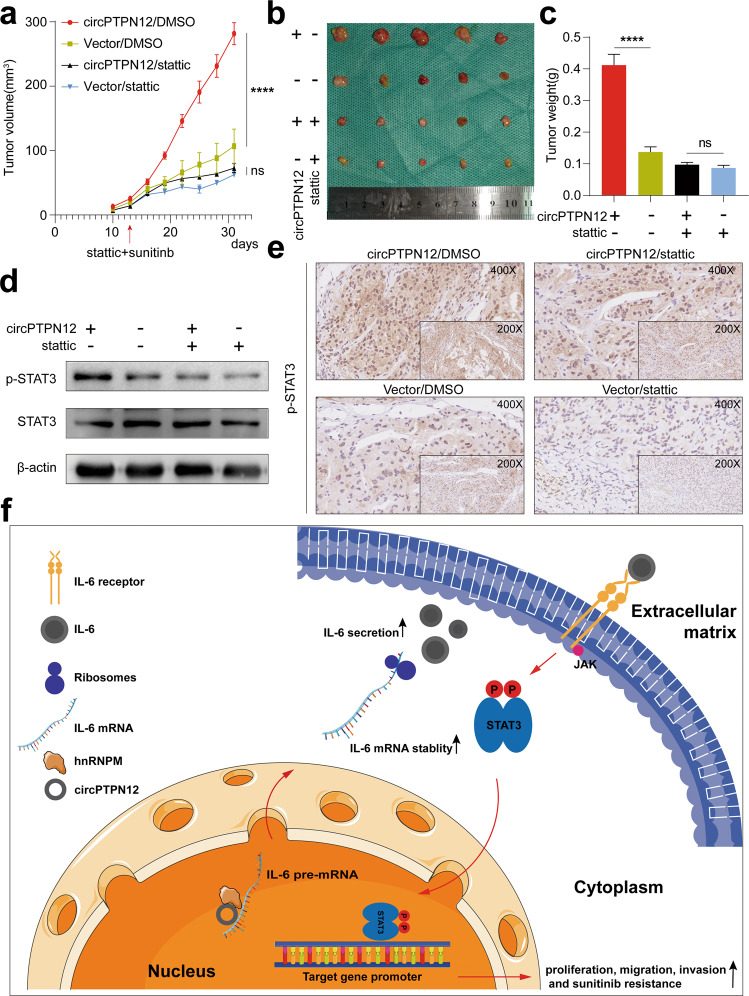
circPTPN12 accelerates tumor growth and sunitinib resistance in vivo. **a** The effect of circPTPN12 and stattic on the growth curve of subcutaneous tumors. **b** The effect of circPTPN12 and stattic on the subcutaneous tumors in nude mice. **c** The weight of subcutaneous tumors was measured after the mice were killed. **d** The p-STAT3 levels in subcutaneous tumors were measured using Western blot. **e** IHC staining of p-STAT3 in different group of subcutaneous tumors. **f** Proposed model illustrating the mechanism by which circPTPN12 promoted the progression and sunitinib resistance of RCC. ns: not significant, ****P < 0.0001. The results were expressed with mean ± SD. All the experiments were replicated three times.

To further investigate whether circPTPN12 could promote tumor growth and sunitinib resistance in nude mice by activating the STAT3 signaling pathway, 786-O cell line that stably overexpressed circPTPN12 and a negative control group were constructed and implanted subcutaneously into nude mice. Since previous results had shown that circPTPN12 accelerated sunitinib resistance of RCC cells via STAT3 pathway, we further investigated whether stattic could act as a small-molecule drug to alleviate the sunitinib resistance caused by circPTPN12 overexpression. Each group was divided into two equal groups based on whether subsequent stattic intraperitoneal injections were given. All mice were treated with sunitinib by gavage daily and the tumor sizes were measured every three days after the formation of tumors. We found that the growth rates of subcutaneous tumors in the control group slowed down significantly after stattic and sunitinib treatment. And the growth rates of tumors in which circPTPN12 was overexpressed were not significantly affected after sunitinib treatment, while the stattic therapy on this basis effectively slowed down the growth of the tumors (Fig. 8a–c). IHC staining and western blot were next performed to detect the phosphorylation levels of STAT3 in tumor tissues, and the results were consistent with the tumor growth curve (Fig. 8d, e). These results showed that circPTPN12 accelerated tumor growth and sunitinib resistance via the STAT3 pathway and stattic combined with sunitinib therapy reduced the growth rate and sunitinib resistance of tumors in vivo.

Overall, we suggest a model in which circPTPN12/hnRNPM complex interacts with IL-6 pre-mRNA and improves its stability, then further activates the STAT3 pathway and promotes the progression and sunitinib resistance of RCC (Fig. 8f).

## Discussion

In this study, we revealed that circPTPN12 played an important role in RCC progression and sunitinib resistance. First, we found that circPTPN12 was highly expressed in RCC and was associated with a poorer prognosis. Further transcriptome sequencing revealed that circPTPN12 affected the sunitinib resistance of RCC cells mainly by regulating the STAT3 signaling pathway. Mechanistically, circPTPN12 formed circRNA-protein complex with hnRNPM in the nucleus and enhanced the ability of hnRNPM to stabilize IL-6 pre-mRNA, thereby further activating the STAT3 pathway and ultimately promoting the progression and sunitinib resistance of RCC.

We first investigated the signaling pathways affected by circPTPN12 using transcriptome sequencing and KEGG enrichment analysis. JAK/STAT pathway was found to be the most significantly altered pathway and we further verified that the level of phosphorylated STAT3 increased, which raised the question of how circPTPN12 affects STAT3 phosphorylation when they have different subcellular localizations. By reviewing the literature, we assumed that circPTPN12 activated the STAT3 pathway by regulating its upstream molecules. And the study by Xu et al. gave a theoretical basis to our hypothesis [38]. Finally, we found circPTPN12 promoted the progression and sunitinib resistance of RCC cells through IL-6/STAT3 pathway.

Recent studies have identified multiple regulatory mechanisms through which circRNAs influence the progression, metabolism, immunity, and therapeutic resistance of cancers [39]. In addition to serving as miRNA sponges, circRNAs are also involved in the regulation of signaling pathways through binding with proteins and translating into functional peptides [40–42]. Previous studies have shown that circPTPN12 is associated with the regulation of epithelial cell fibrosis [19, 20]. However, the location of circPTPN12 was not discussed in these studies, which had a decisive influence on the regulatory mechanism of circRNAs. In present study, we investigated the subcellular localization of circPTPN12 by RNA cellular fractionation assay and FISH experiments, thus we verified that circPTPN12 was mainly distributed in the nucleus. Besides, subsequent protein mass spectrometry analysis didn’t find evidence for the binding between circPTPN12 and AGO2, a vital protein through which circRNAs could serve as miRNA sponges [33, 34]. These findings convinced us that ceRNA is not the primary regulatory mechanism of circPTPN12 in RCC.

hnRNPM was identified as the protein that directly interacted with circPTPN12. hnRNPM is a protein that has not been studied deeply and reports concerning its role in drug resistance are rare. Studies on its biological functions are mainly focused on alternative splicing [43, 44]. For example, Wang et al. found that circURI1 interacted with hnRNPM to modulate the alternative splicing of VEGFA and further suppressed the metastasis of gastric cancer [45]. Some recent studies have also shown that hnRNPM is involved in regulating the stability of target mRNA. Chen et al. demonstrated that the binding of hnRNPM and its target mRNA FGF9 enhanced FGF9 mRNA stability and promoted translation initiation [46]. We first verified whether hnRNPM regulated IL-6 levels through alternative splicing. However, all isoforms of IL-6 decreased including RCC inhibitor IL-6 isoform 2, which suggested the level of IL-6 was not regulated by hnRNPM-mediated alternative splicing. Further Actinomycin D assays and rescue assays showed circPTPN12/hnRNPM regulated the rate of degradation of IL-6 mRNA and the activation of the STAT3 pathway.

As a commonly used first-line drug in clinical practice, the use of sunitinib has greatly improved the prognosis of RCC patients [47]. However, As the course of treatment progresses, the efficacy of sunitinib is greatly reduced by the development of adaptive drug resistance [48, 49]. In our research, we found the tumors overexpressing circPTPN12 were insensitive to sunitinib treatment. However, when treated with a combination of sunitinib and stattic, the growth rate of tumors slowed down significantly, which might provide a new combination regimen for relieving sunitinib resistance.

In summary, we proposed and validated the mechanistic hypothesis that circPTPN12 activated the hnRNPM-mediated IL-6/STAT3 pathway and therefore promoted the progression and sunitinib resistance of RCC. Our findings could provide a potential therapeutic target for sunitinib-resistant RCC.

## Supplementary information

FigS1

Supplementary Figure Legends

Supplementary table1 to 5

Supplementary table 6

Protein gel original data

checklist

## Data Availability

The data that support the findings of this study are available upon reasonable request.

## References

[1] Hsieh JJ, Purdue MP, Signoretti S, Swanton C, Albiges L, Schmidinger M (2017). Renal cell carcinoma. Nat Rev Dis Prim.

[2] Moch H, Cubilla AL, Humphrey PA, Reuter VE, Ulbright TM (2016). The 2016 WHO classification of tumours of the urinary system and male genital organs—Part A: renal, penile, and testicular tumours. Eur Urol.

[3] Siegel RL, Miller KD, Fuchs HE, Jemal A (2021). Cancer statistics, 2021. CA: Cancer J Clin.

[4] Cohen HT, McGovern FJ (2005). Renal-cell carcinoma. N. Engl J Med.

[5] Escudier B, Szczylik C, Porta C, Gore M (2012). Treatment selection in metastatic renal cell carcinoma: expert consensus. Nat Rev Clin Oncol.

[6] Kanesvaran R, Porta C, Wong A, Powles T, Ng QS, Schmidinger M (2021). Pan-Asian adapted ESMO Clinical Practice Guidelines for the diagnosis, treatment and follow-up of patients with renal cell carcinoma. ESMO Open.

[7] Molina AM, Lin X, Korytowsky B, Matczak E, Lechuga MJ, Wiltshire R (2014). Sunitinib objective response in metastatic renal cell carcinoma: analysis of 1059 patients treated on clinical trials. Eur J Cancer.

[8] Bergers G, Hanahan D (2008). Modes of resistance to anti-angiogenic therapy. Nat Rev Cancer.

[9] Kristensen LS, Andersen MS, Stagsted LVW, Ebbesen KK, Hansen TB, Kjems J (2019). The biogenesis, biology and characterization of circular RNAs. Nat Rev Genet.

[10] Liu L, Gu M, Ma J, Wang Y, Li M, Wang H (2022). CircGPR137B/miR-4739/FTO feedback loop suppresses tumorigenesis and metastasis of hepatocellular carcinoma. Mol Cancer.

[11] Yu L, Zhu H, Wang Z, Huang J, Zhu Y, Fan G (2022). Circular RNA circFIRRE drives osteosarcoma progression and metastasis through tumorigenic-angiogenic coupling. Mol Cancer.

[12] Zhang LX, Gao J, Long X, Zhang PF, Yang X, Zhu SQ (2022). The circular RNA circHMGB2 drives immunosuppression and anti-PD-1 resistance in lung adenocarcinomas and squamous cell carcinomas via the miR-181a-5p/CARM1 axis. Mol Cancer.

[13] Hu C, Xia R, Zhang X, Li T, Ye Y, Li G (2022). circFARP1 enables cancer-associated fibroblasts to promote gemcitabine resistance in pancreatic cancer via the LIF/STAT3 axis. Mol Cancer.

[14] Wang S, Wang Y, Li Q, Li X, Feng X (2022). A novel circular RNA confers trastuzumab resistance in human epidermal growth factor receptor 2-positive breast cancer through regulating ferroptosis. Environ Toxicol.

[15] Li Y, Yang X, Xiong X (2022). Circ_0004015 silencing represses cisplatin chemoresistance and tumor progression by reducing KLF8 in a miR-198-dependent manner in non-small cell lung cancer. Genomics.

[16] Chen H, Li F, Xue Q (2022). Circ-CUL2/microRNA-888-5p/RB1CC1 axis participates in cisplatin resistance in NSCLC via repressing cell advancement. Bioengineered.

[17] Tan L, Huang Z, Chen Z, Chen S, Ye Y, Chen T (2023). CircRNA_001895 promotes sunitinib resistance of renal cell carcinoma through regulation of apoptosis and DNA damage repair. J Chemother.

[18] Huang KB, Pan YH, Shu GN, Yao HH, Liu X, Zhou M (2021). Circular RNA circSNX6 promotes sunitinib resistance in renal cell carcinoma through the miR-1184/GPCPD1/ lysophosphatidic acid axis. Cancer Lett.

[19] Song M, Zhao G, Sun H, Yao S, Zhou Z, Jiang P (2021). circPTPN12/miR-21-5 p/∆Np63α pathway contributes to human endometrial fibrosis. eLife.

[20] Liu F, Li T, Zhan X (2022). Silencing circular RNAPTPN12 promoted the growth of keloid fibroblasts by activating Wnt signaling pathway via targeting microRNA-21-5p. Bioengineered.

[21] Xiong W, Zhang B, Yu H, Zhu L, Yi L, Jin X (2021). RRM2 regulates sensitivity to sunitinib and PD-1 blockade in renal cancer by stabilizing ANXA1 and activating the AKT pathway. Adv Sci.

[22] Xiao C, Zhang W, Hua M, Chen H, Yang B, Wang Y (2022). RNF7 inhibits apoptosis and sunitinib sensitivity and promotes glycolysis in renal cell carcinoma via the SOCS1/JAK/STAT3 feedback loop. Cell Mol Biol Lett.

[23] Zhao T, Bao Y, Gan X, Wang J, Chen Q, Dai Z (2019). DNA methylation-regulated QPCT promotes sunitinib resistance by increasing HRAS stability in renal cell carcinoma. Theranostics.

[24] Huynh J, Chand A, Gough D, Ernst M (2019). Therapeutically exploiting STAT3 activity in cancer - using tissue repair as a road map. Nat Rev Cancer.

[25] Johnson DE, O’Keefe RA, Grandis JR (2018). Targeting the IL-6/JAK/STAT3 signalling axis in cancer. Nat Rev Clin Oncol.

[26] Yu H, Lee H, Herrmann A, Buettner R, Jove R (2014). Revisiting STAT3 signalling in cancer: new and unexpected biological functions. Nat Rev Cancer.

[27] Pore N, Wu S, Standifer N, Jure-Kunkel M, de Los Reyes M, Shrestha Y (2021). Resistance to durvalumab and durvalumab plus tremelimumab is associated with functional STK11 mutations in patients with non-small cell lung cancer and is reversed by STAT3 knockdown. Cancer Discov.

[28] Hu B, Zou T, Qin W, Shen X, Su Y, Li J (2022). Inhibition of EGFR overcomes acquired lenvatinib resistance driven by STAT3-ABCB1 signaling in hepatocellular carcinoma. Cancer Res.

[29] Sun Y, Dong Y, Liu X, Zhang Y, Bai H, Duan J (2022). Blockade of STAT3/IL-4 overcomes EGFR T790M-cis-L792F-induced resistance to osimertinib via suppressing M2 macrophages polarization. EBioMedicine.

[30] Latourte A, Cherifi C, Maillet J, Ea HK, Bouaziz W, Funck-Brentano T (2017). Systemic inhibition of IL-6/Stat3 signalling protects against experimental osteoarthritis. Ann Rheum Dis.

[31] McMurray JS (2006). A new small-molecule Stat3 inhibitor. Chem Biol.

[32] Yu TJ, Liu YY, Li XG, Lian B, Lu XX, Jin X (2021). PDSS1-mediated activation of CAMK2A-STAT3 signaling promotes metastasis in triple-negative breast cancer. Cancer Res.

[33] Marzec M (2020). New insights into the function of mammalian Argonaute2. PLoS Genet.

[34] Hutvagner G, Simard MJ (2008). Argonaute proteins: key players in RNA silencing. Nat Rev Mol Cell Biol.

[35] Nazer E, Gómez Acuña L, Kornblihtt AR (2022). Seeking the truth behind the myth: Argonaute tales from “nuclearland”. Mol Cell.

[36] Alberti L, Bachelot T, Duc A, Biota C, Blay JY (2005). A spliced isoform of interleukin 6 mRNA produced by renal cell carcinoma encodes for an interleukin 6 inhibitor. Cancer Res.

[37] Cao P, Luo WW, Li C, Tong Z, Zheng ZQ, Zhou L (2019). The heterogeneous nuclear ribonucleoprotein hnRNPM inhibits RNA virus-triggered innate immunity by antagonizing RNA sensing of RIG-I-like receptors. PLoS Pathog.

[38] Xu Z, Yang F, Wei D, Liu B, Chen C, Bao Y (2017). Long noncoding RNA-SRLR elicits intrinsic sorafenib resistance via evoking IL-6/STAT3 axis in renal cell carcinoma. Oncogene.

[39] Kristensen LS, Jakobsen T, Hager H, Kjems J (2022). The emerging roles of circRNAs in cancer and oncology. Nat Rev Clin Oncol.

[40] Zeng Z, Xia L, Fan S, Zheng J, Qin J, Fan X (2021). Circular RNA CircMAP3K5 acts as a MicroRNA-22-3p sponge to promote resolution of intimal hyperplasia via TET2-mediated smooth muscle cell differentiation. Circulation.

[41] Zhang H, Xiao X, Wei W, Huang C, Wang M, Wang L (2021). CircLIFR synergizes with MSH2 to attenuate chemoresistance via MutSα/ATM-p73 axis in bladder cancer. Mol Cancer.

[42] Gao X, Xia X, Li F, Zhang M, Zhou H, Wu X (2021). Circular RNA-encoded oncogenic E-cadherin variant promotes glioblastoma tumorigenicity through activation of EGFR-STAT3 signalling. Nat Cell Biol.

[43] Zhu GQ, Wang Y, Wang B, Liu WR, Dong SS, Chen EB (2022). Targeting HNRNPM Inhibits Cancer Stemness and Enhances Antitumor Immunity in Wnt-activated Hepatocellular Carcinoma. Cell Mol Gastroenterol Hepatol.

[44] Hu X, Harvey SE, Zheng R, Lyu J, Grzeskowiak CL, Powell E (2020). The RNA-binding protein AKAP8 suppresses tumor metastasis by antagonizing EMT-associated alternative splicing. Nat Commun.

[45] Wang X, Li J, Bian X, Wu C, Hua J, Chang S (2021). CircURI1 interacts with hnRNPM to inhibit metastasis by modulating alternative splicing in gastric cancer. Proc Natl Acad Sci USA.

[46] Chen TM, Lai MC, Li YH, Chan YL, Wu CH, Wang YM (2019). hnRNPM induces translation switch under hypoxia to promote colon cancer development. EBioMedicine.

[47] Rini BI, Campbell SC, Escudier B (2009). Renal cell carcinoma. Lancet.

[48] Joosten SC, Hamming L, Soetekouw PM, Aarts MJ, Veeck J, van Engeland M (2015). Resistance to sunitinib in renal cell carcinoma: from molecular mechanisms to predictive markers and future perspectives. Biochim Biophys Acta.

[49] Motzer RJ, Rini BI, Bukowski RM, Curti BD, George DJ, Hudes GR (2006). Sunitinib in patients with metastatic renal cell carcinoma. Jama.

