# Supplementary figures and images for: circPTPN12 promotes the progression and sunitinib resistance of renal cancer via hnRNPM/IL-6/STAT3 pathway
Source: Cell Death Dis. 2023 Mar 31;14(3):232. doi: 10.1038/s41419-023-05717-z (PMC10066201; doi:10.1038/s41419-023-05717-z)

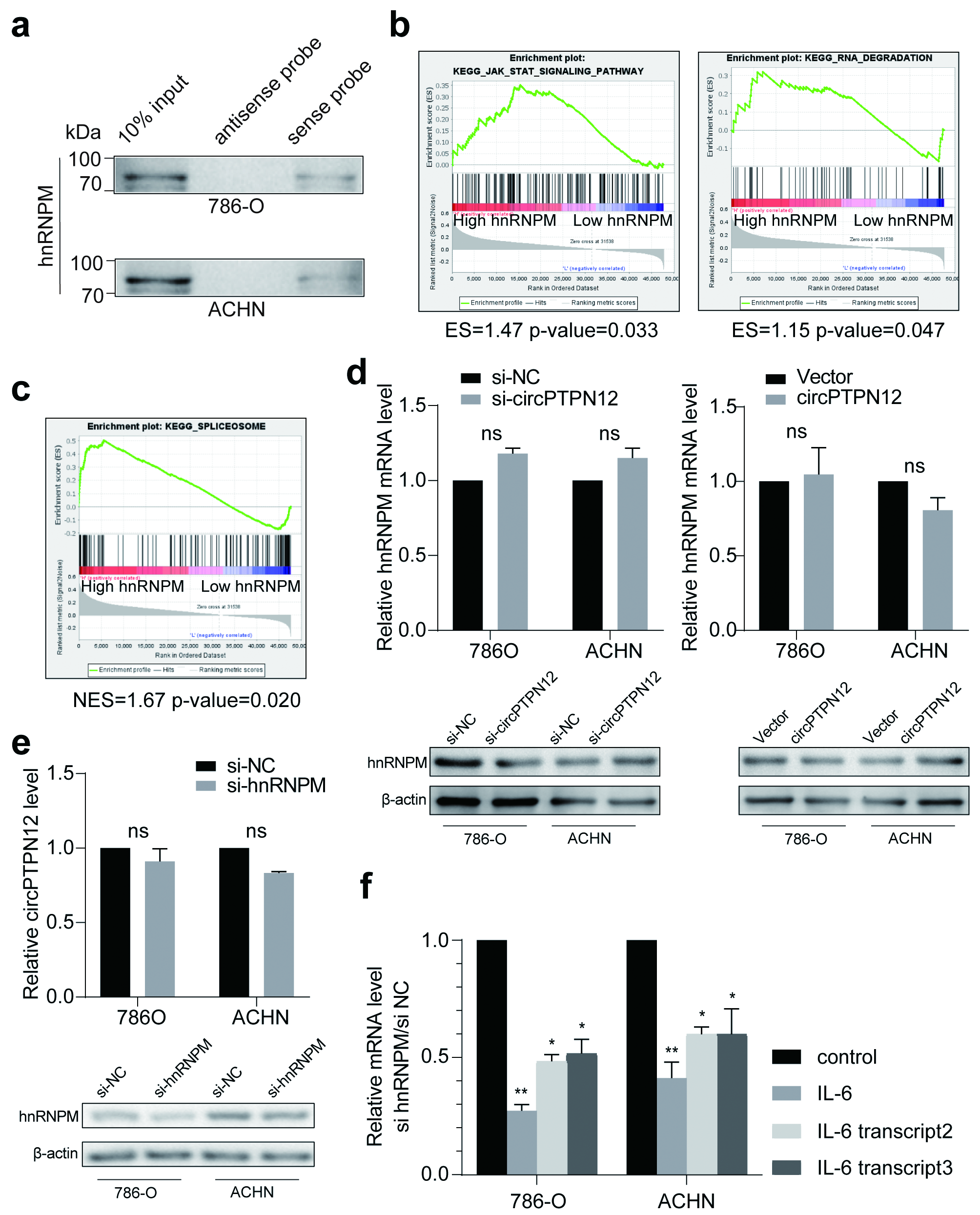

Supplement: Supplementary file 1 — FigS1 [file 41419_2023_5717_MOESM1_ESM.tif]

Fig3D


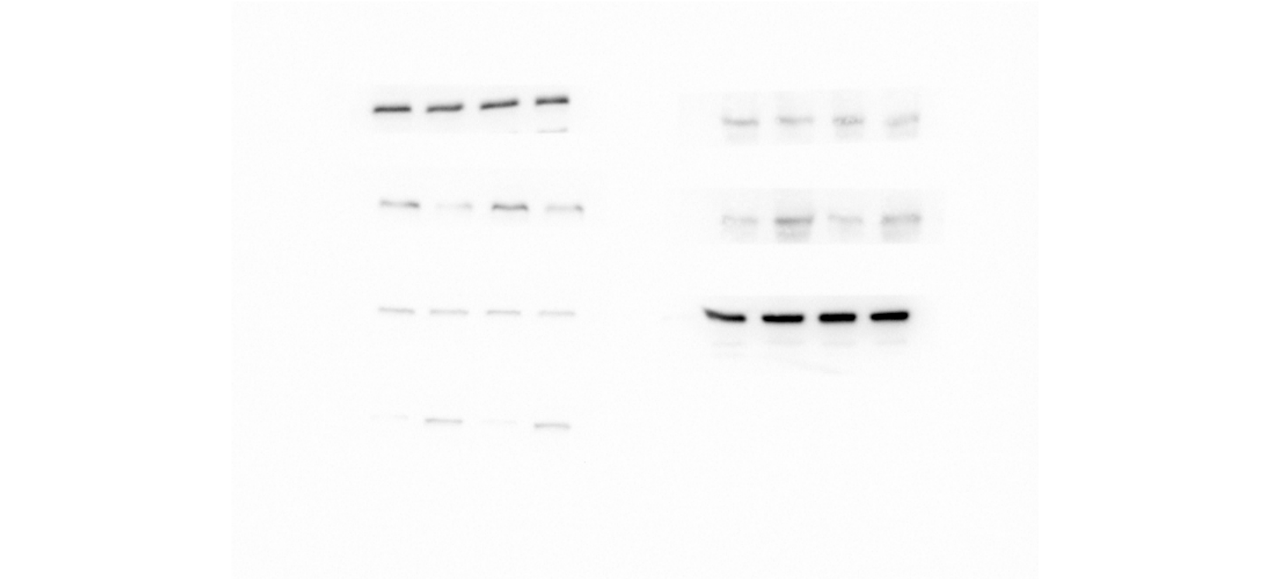

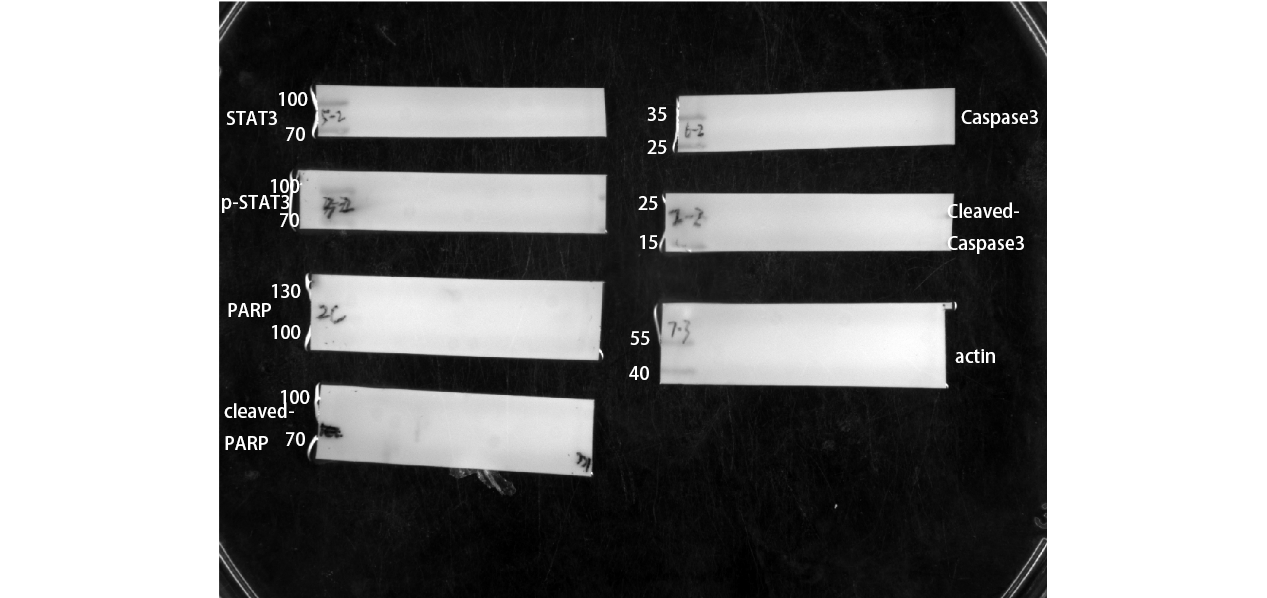


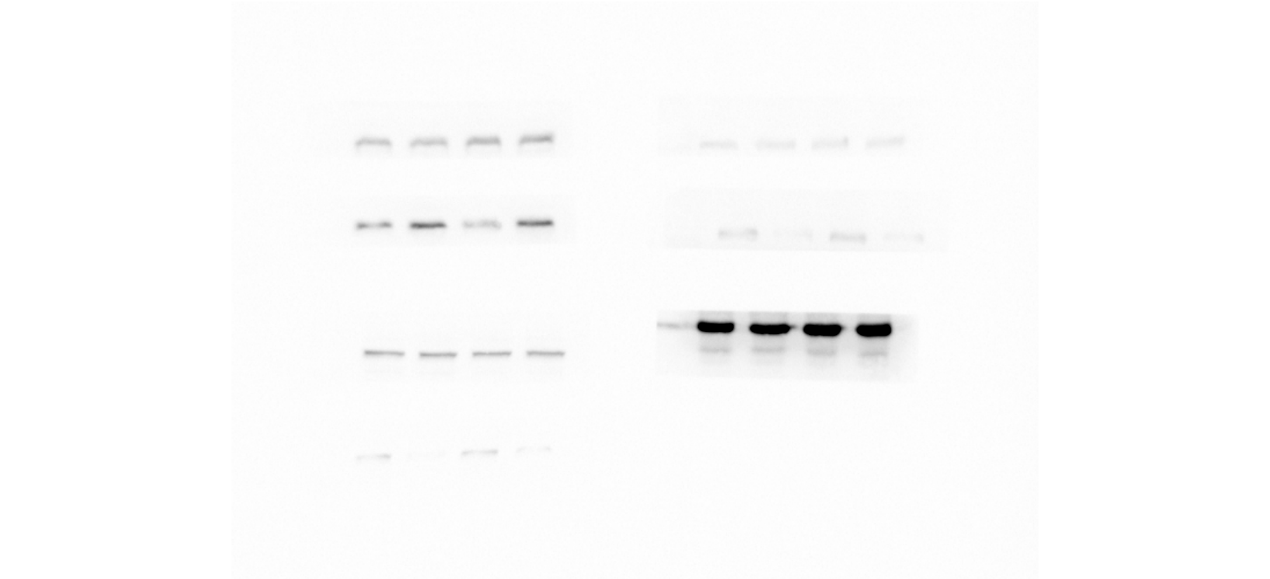

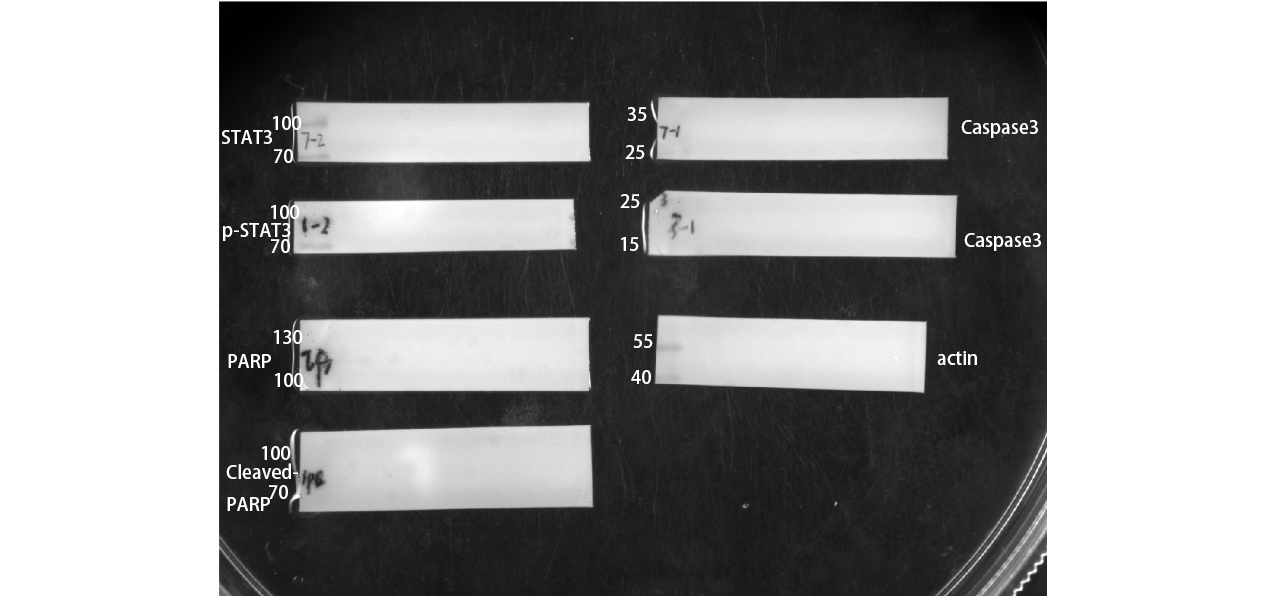


Fig4F


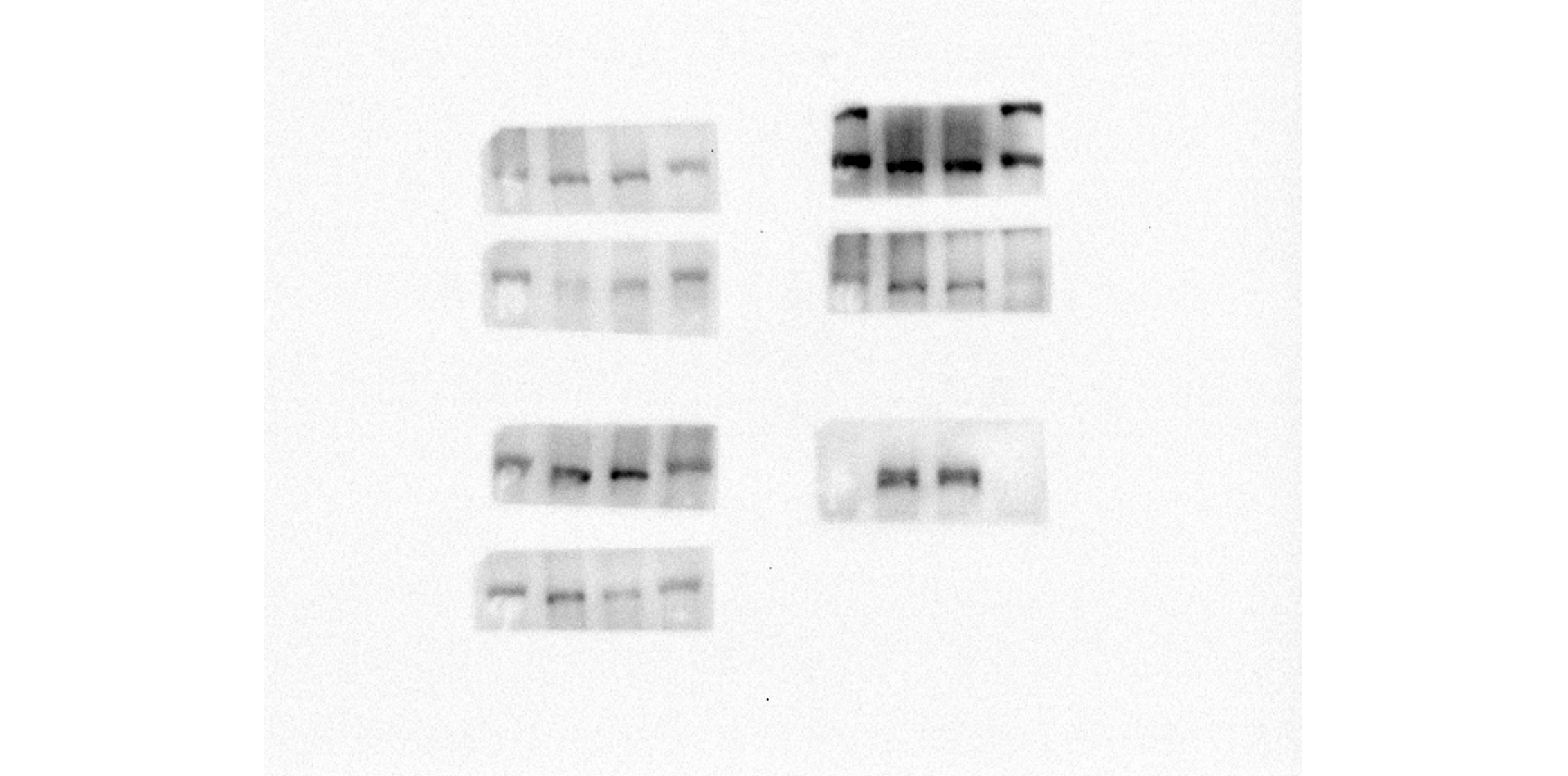


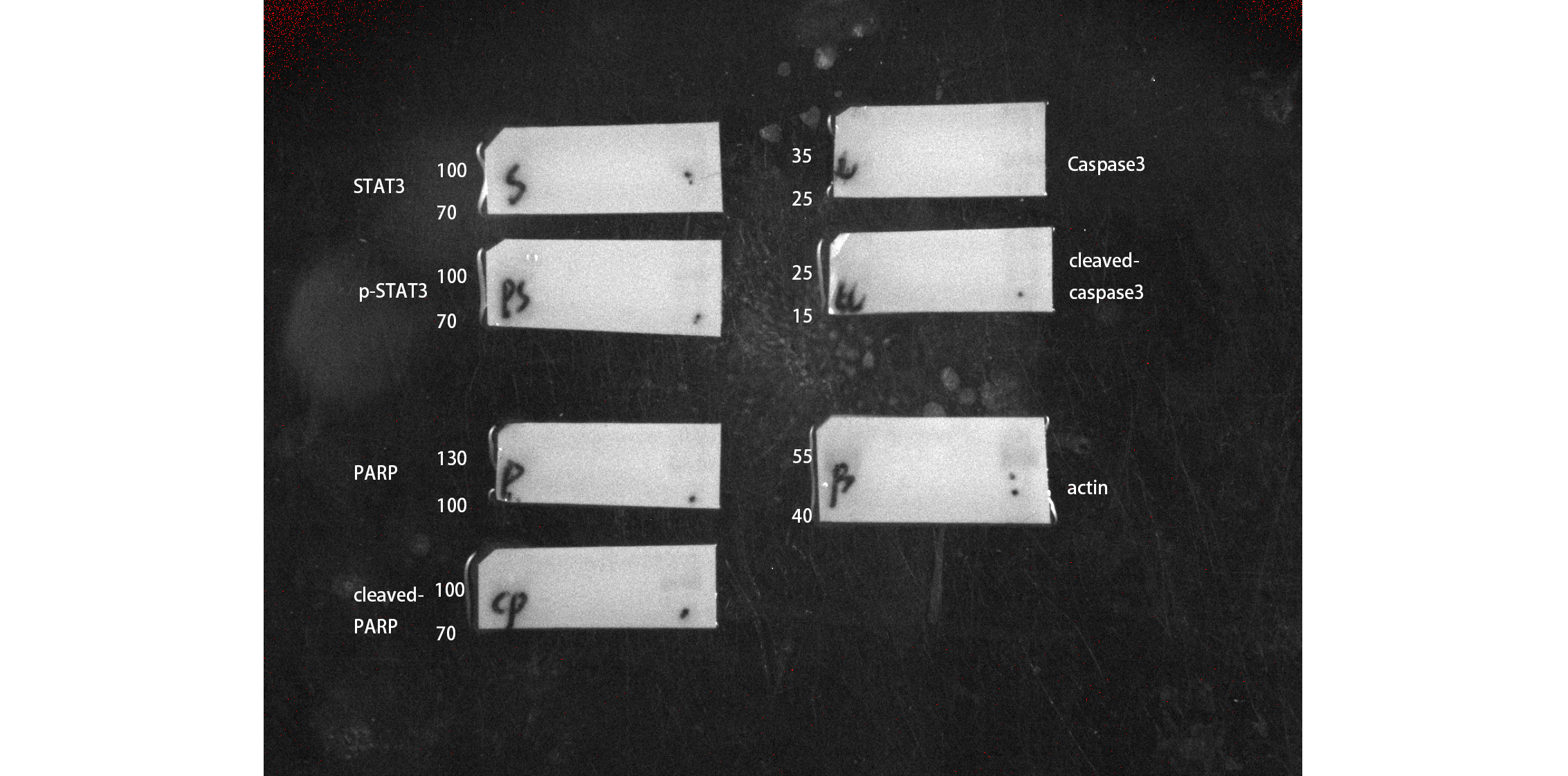


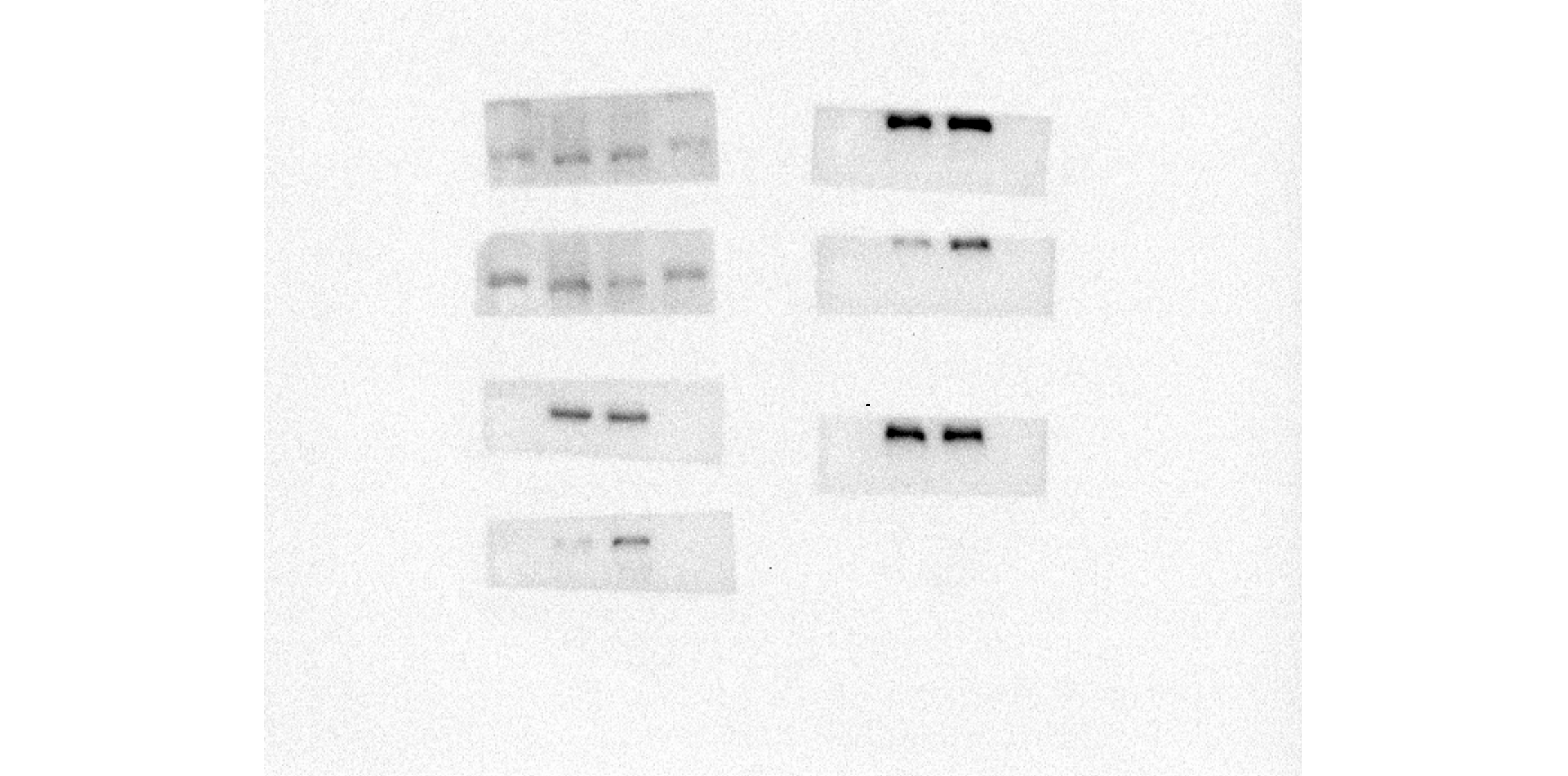

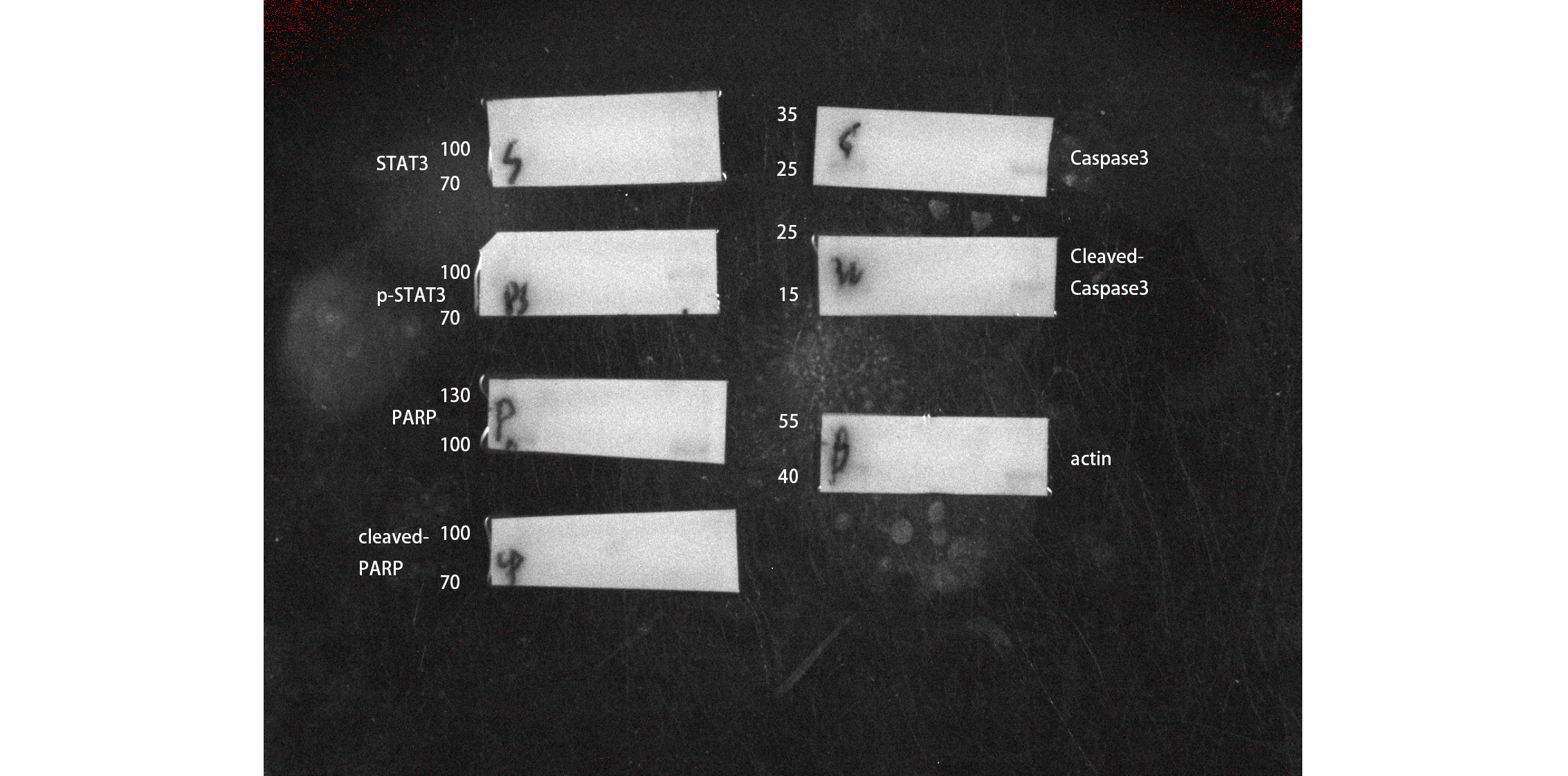


Fig5D and S1A


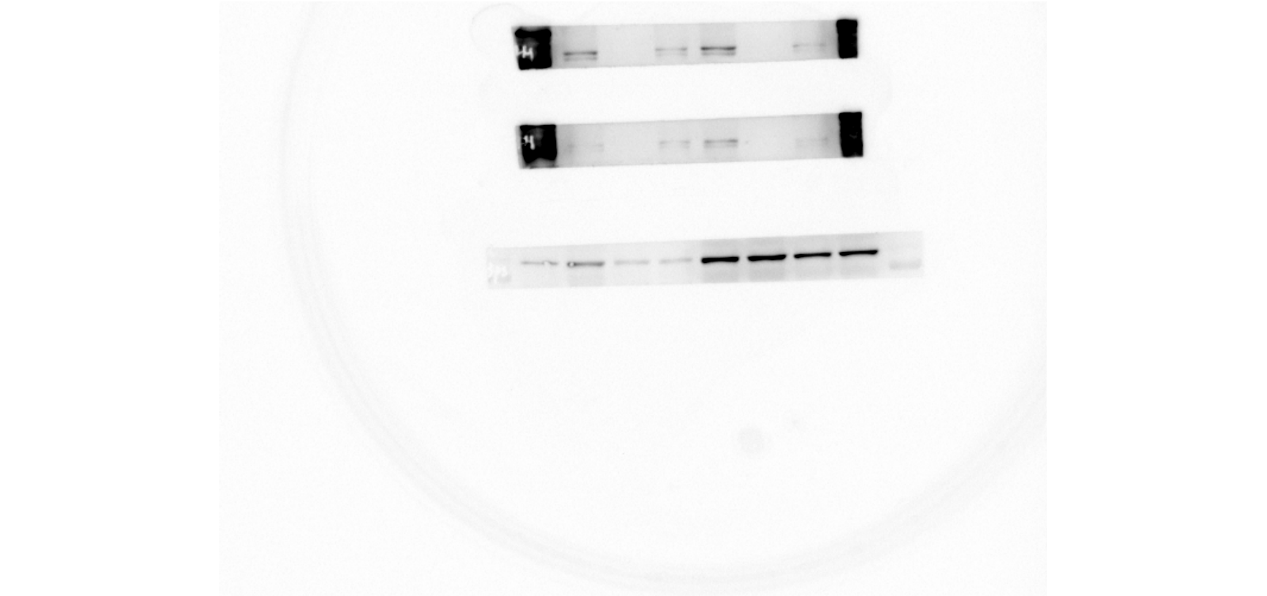

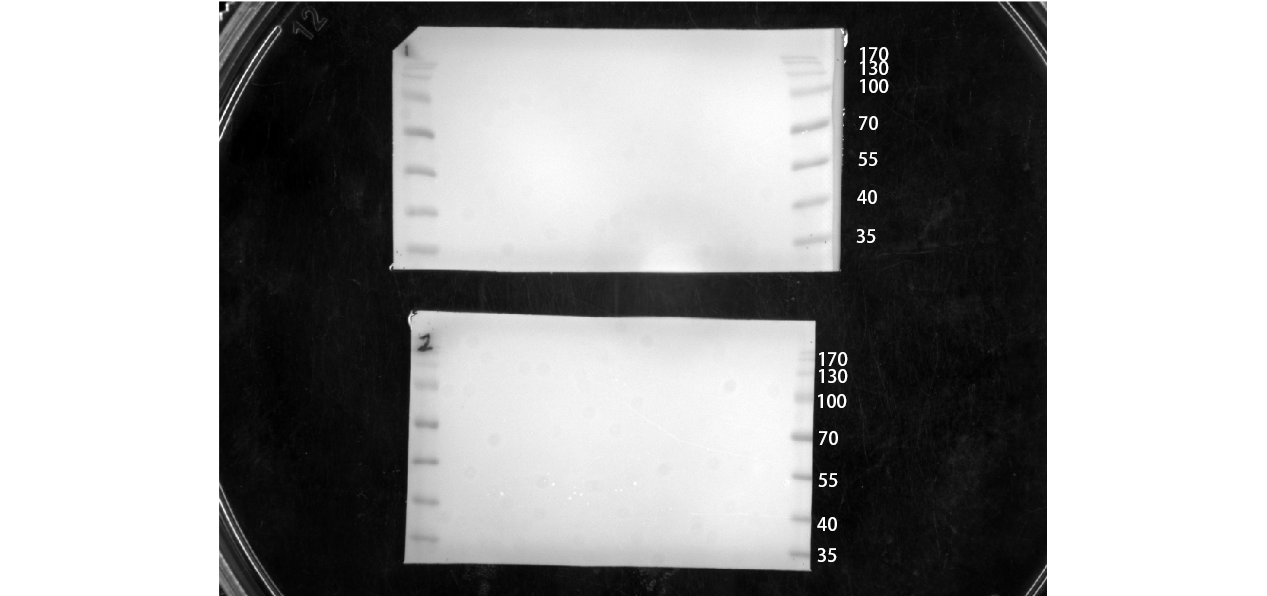


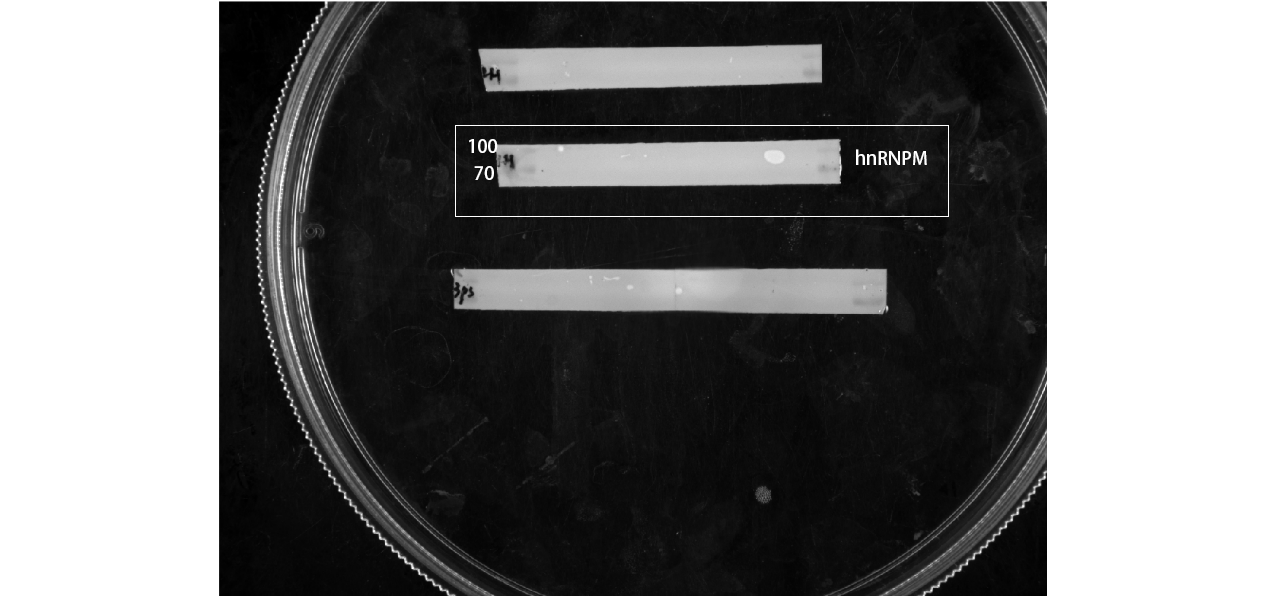


Fig5G


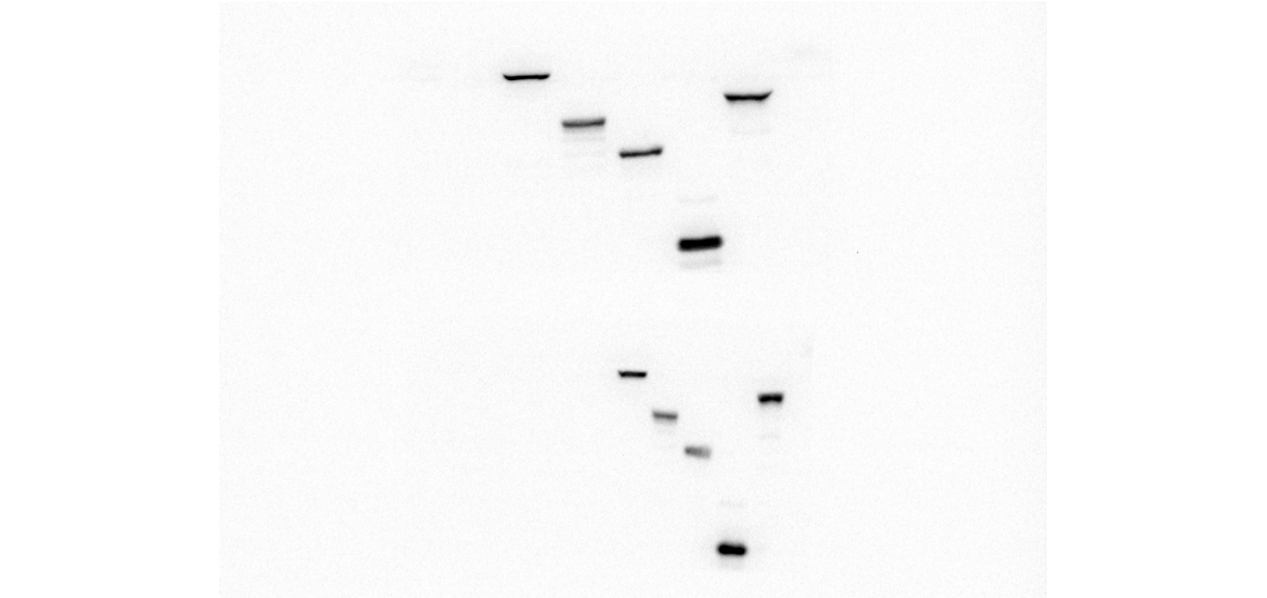

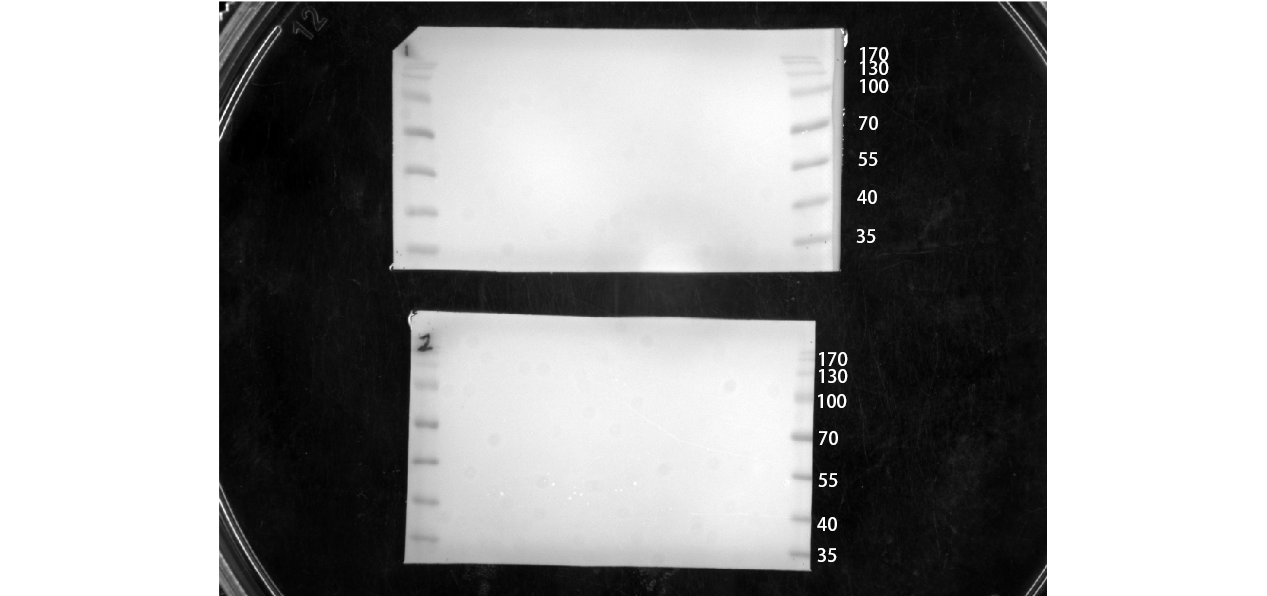


Fig6C


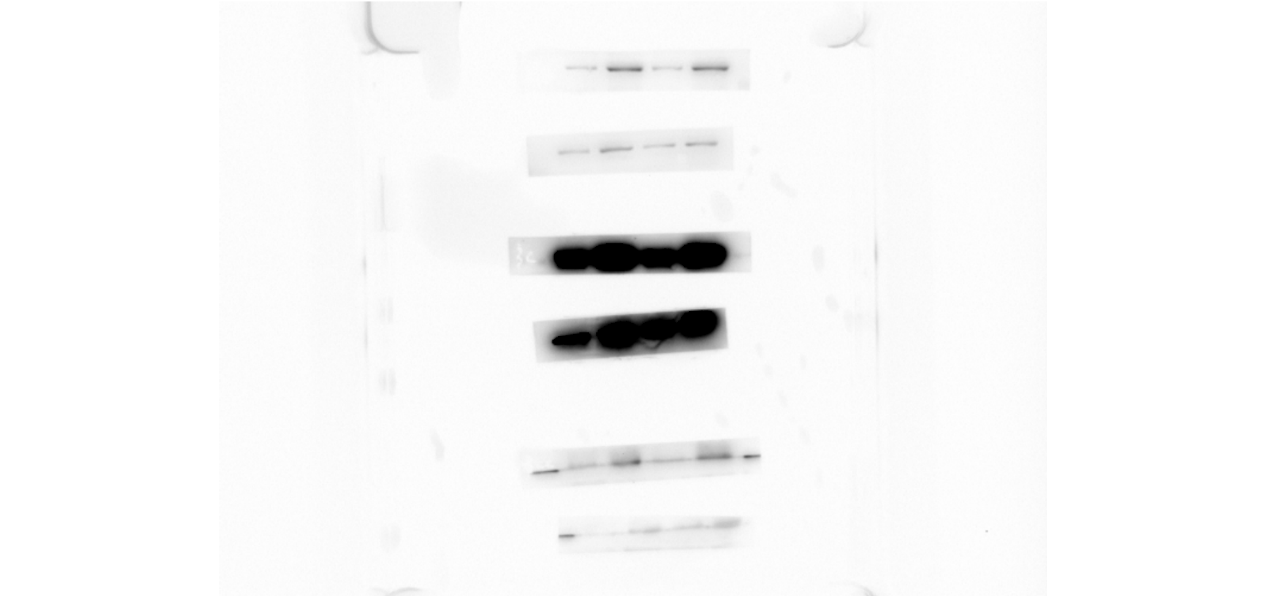

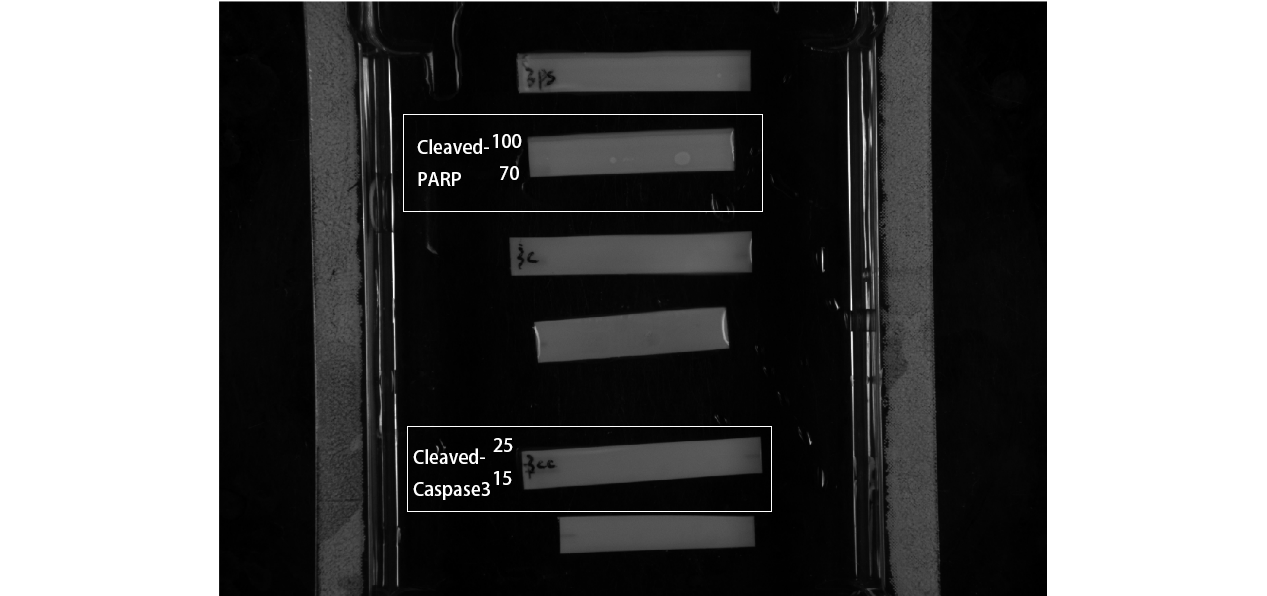


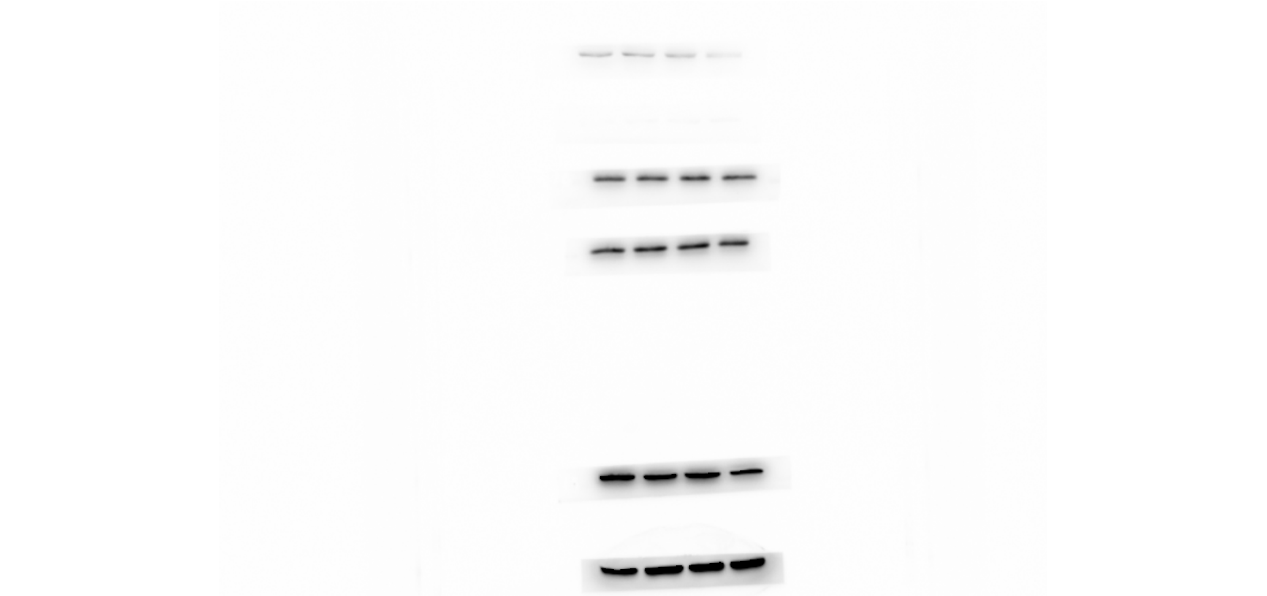

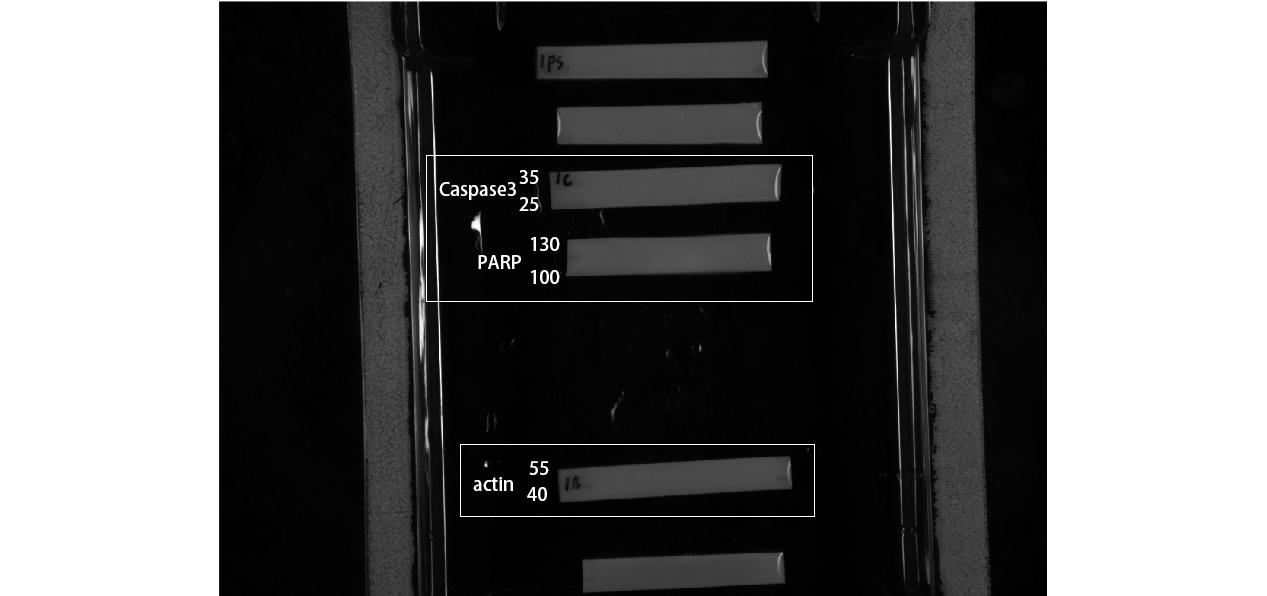


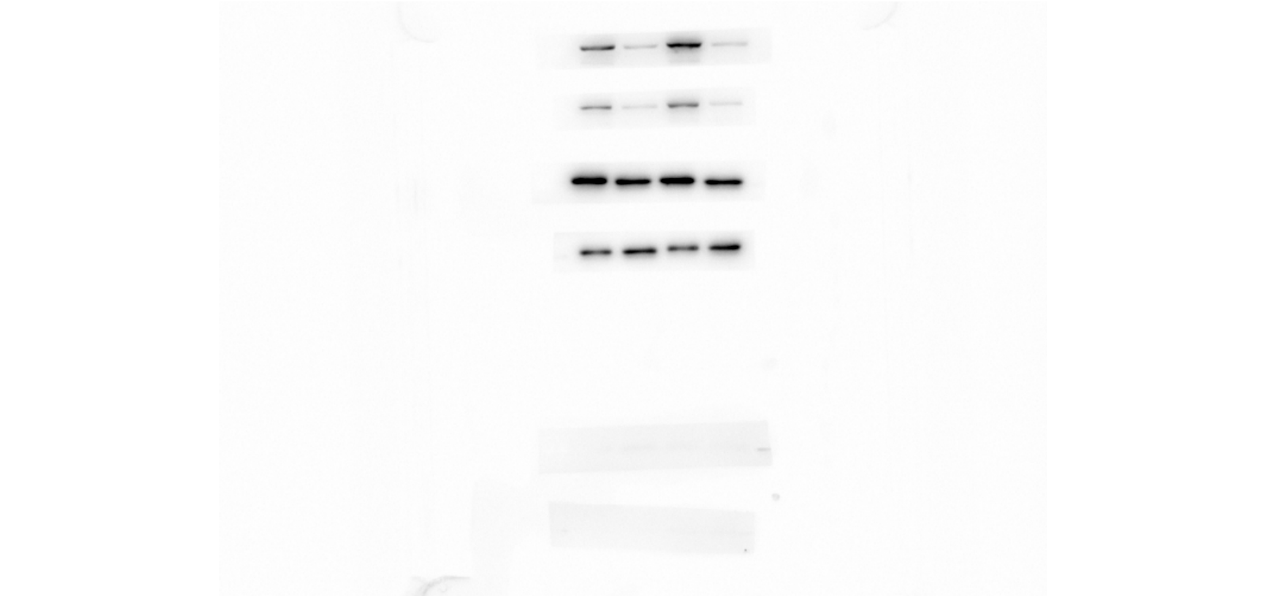


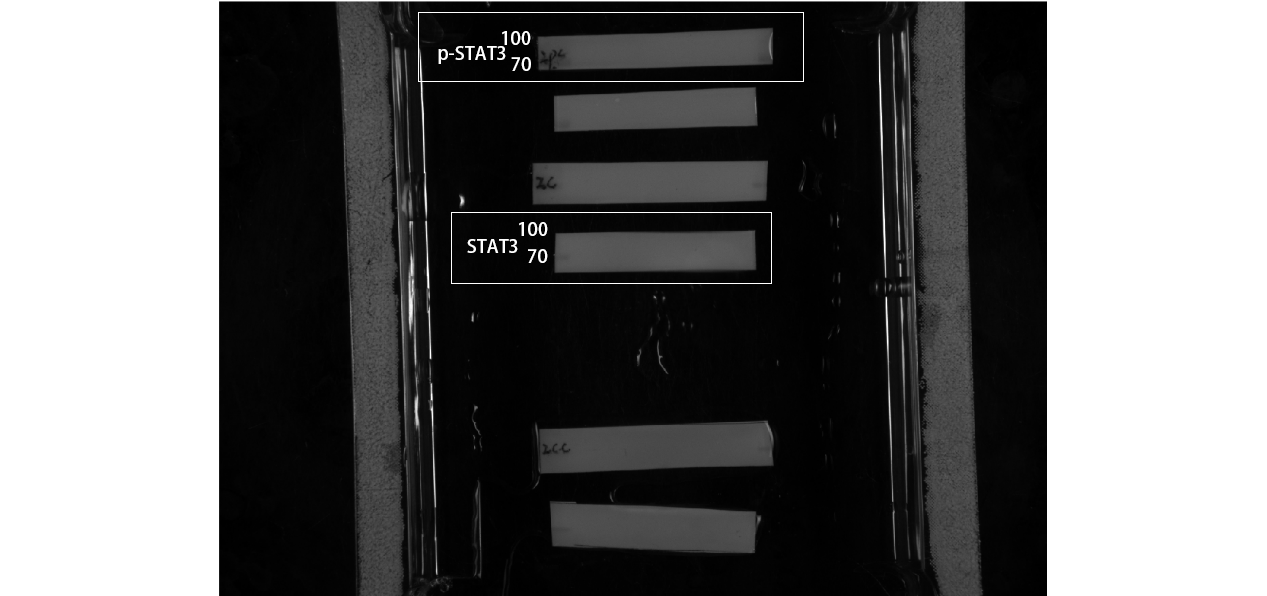


Fig6D


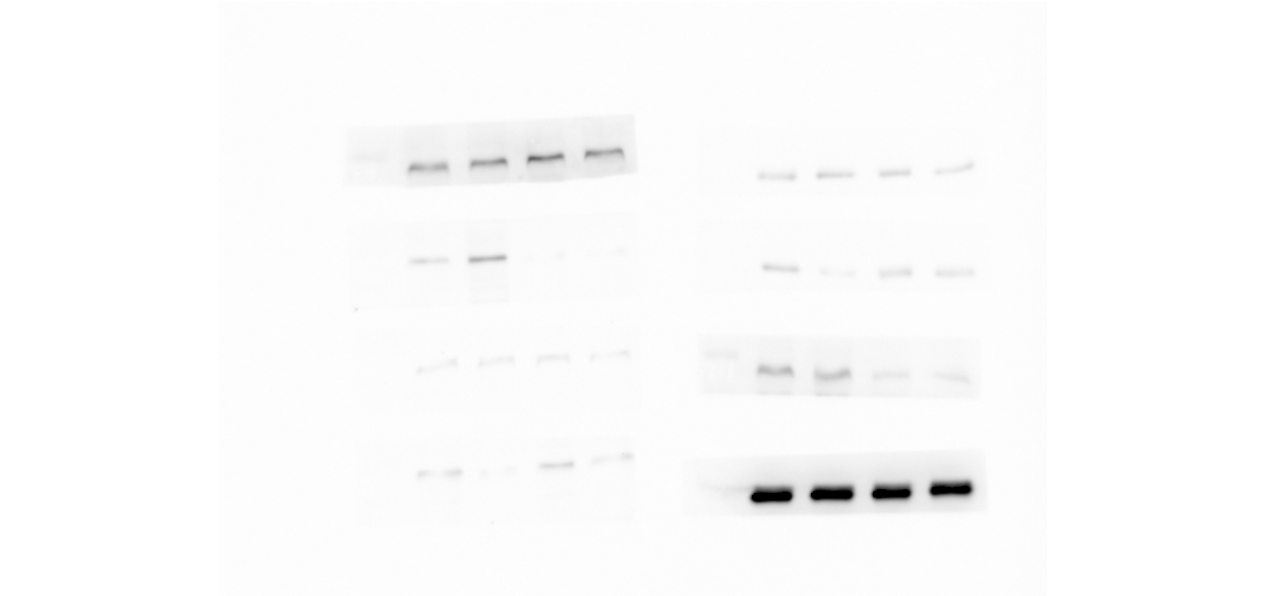


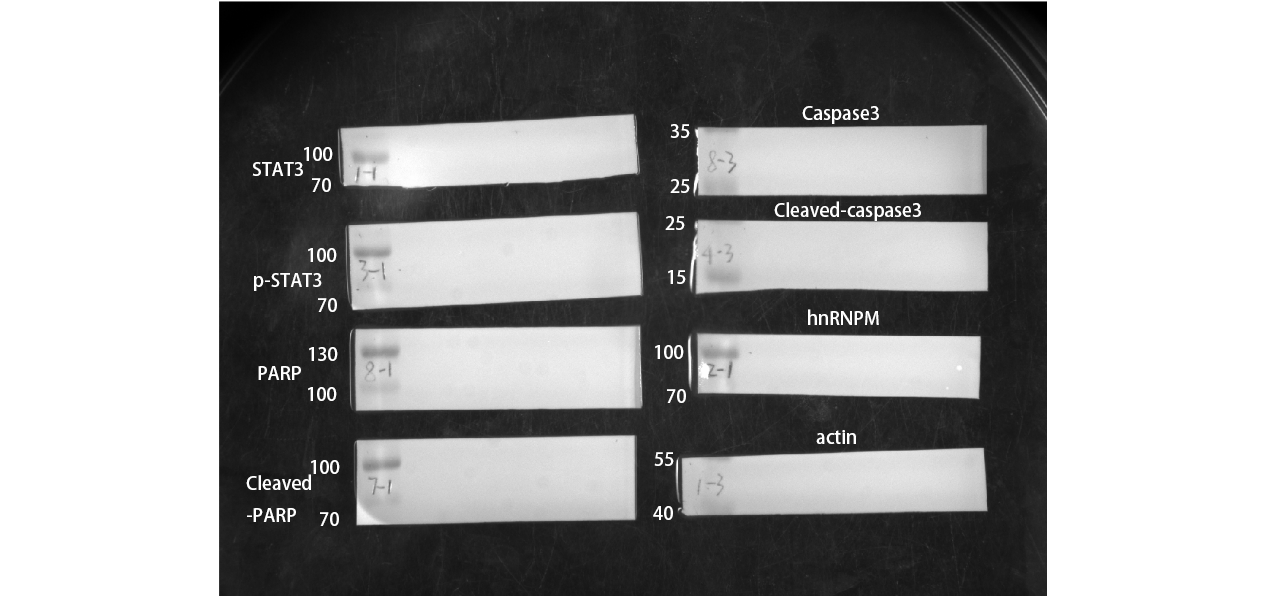


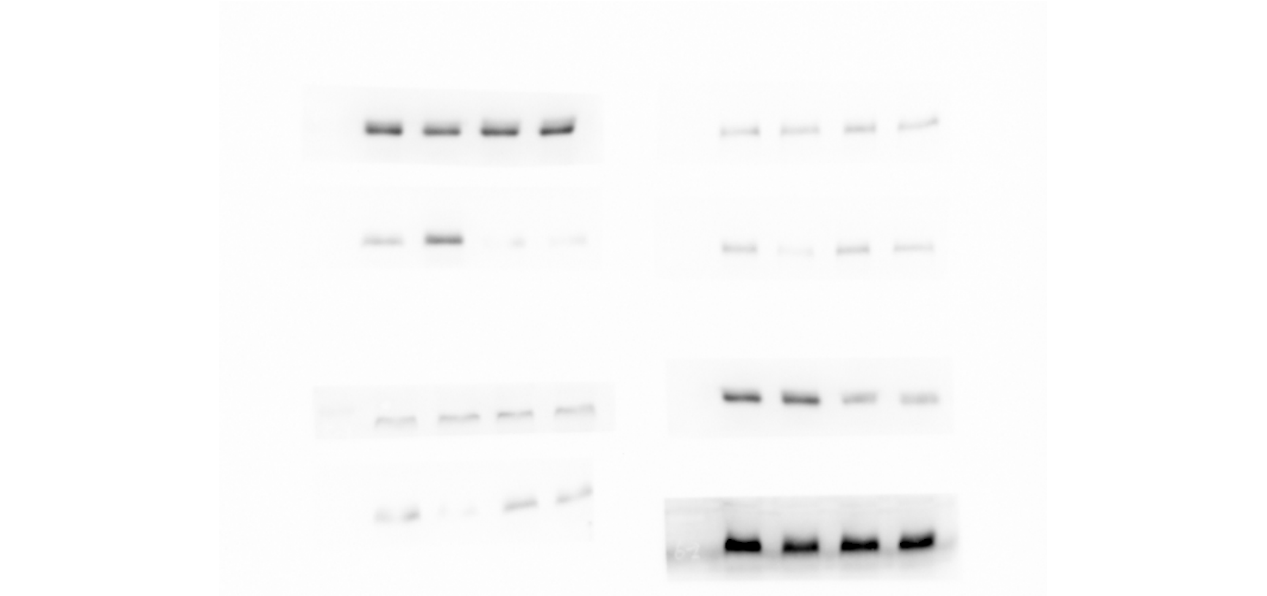


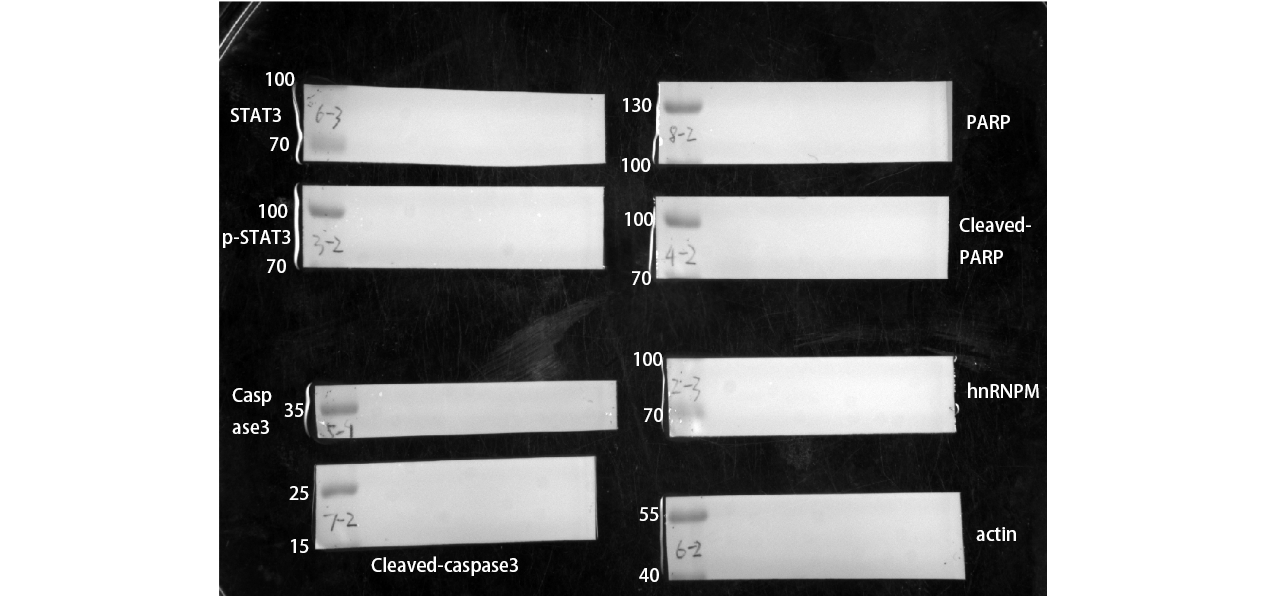


Fig6F


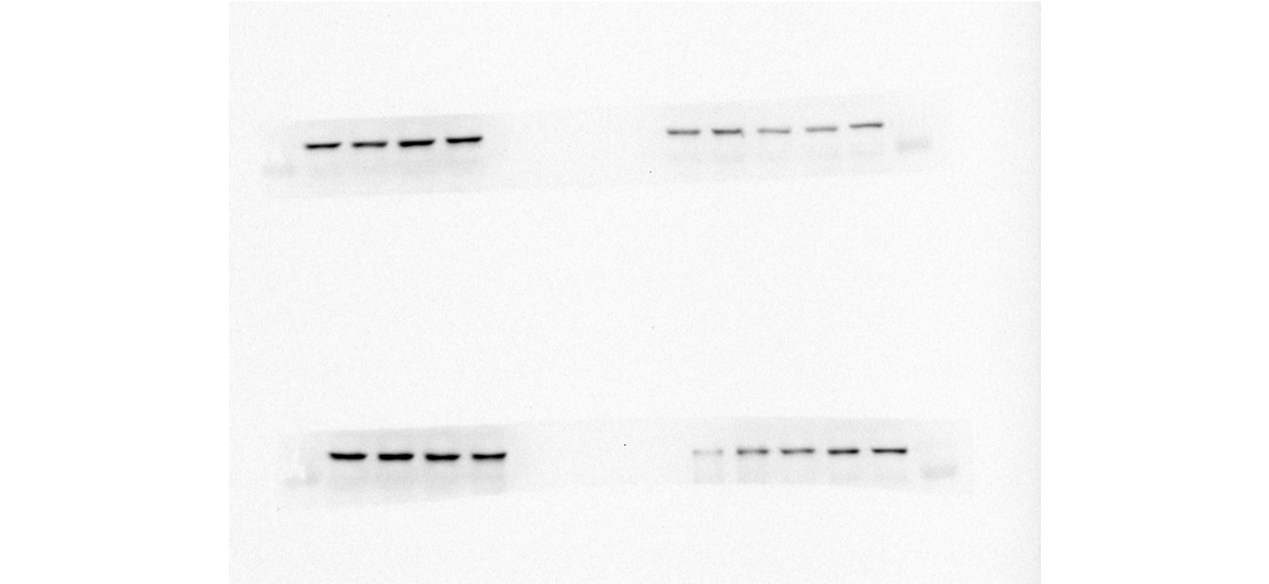


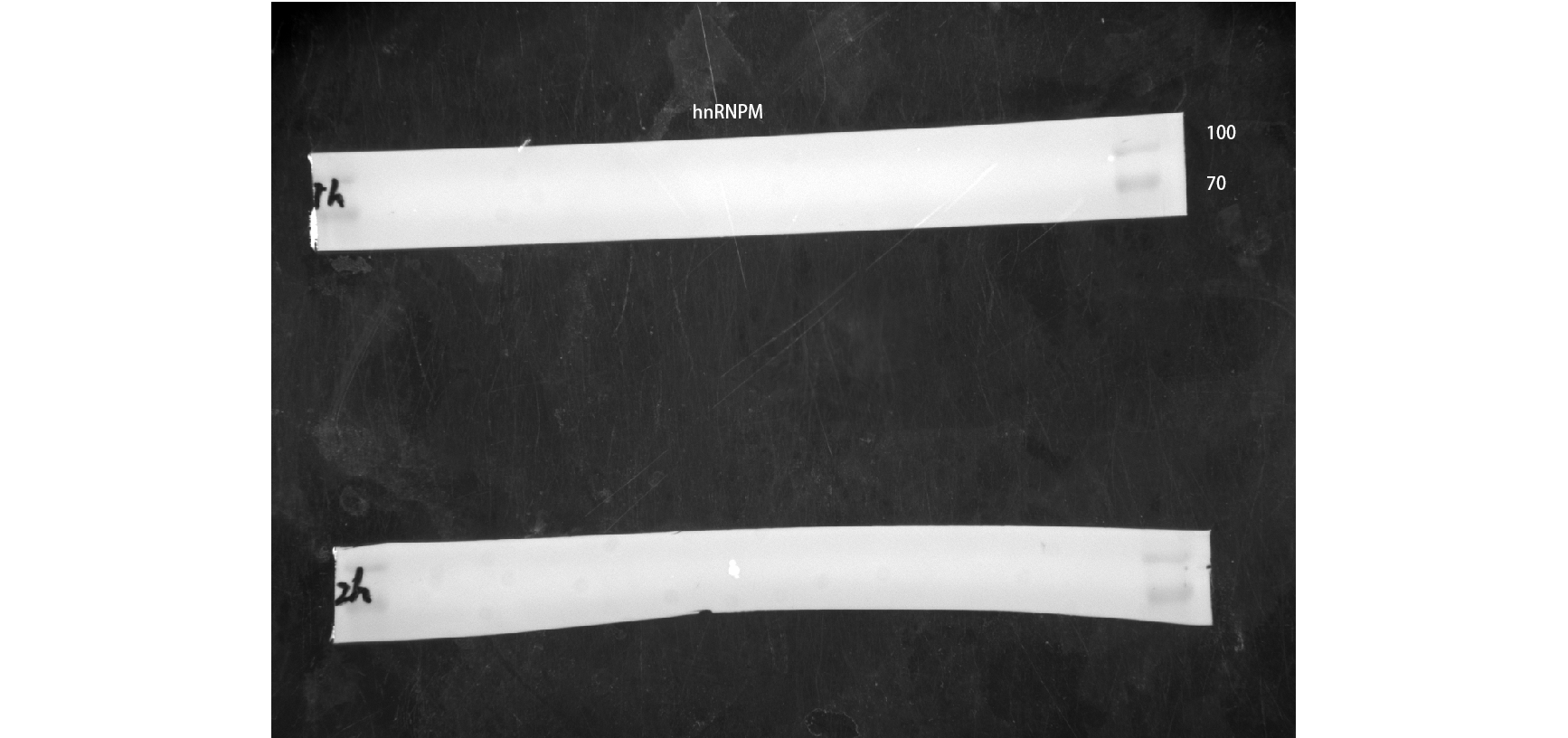


Fig8D


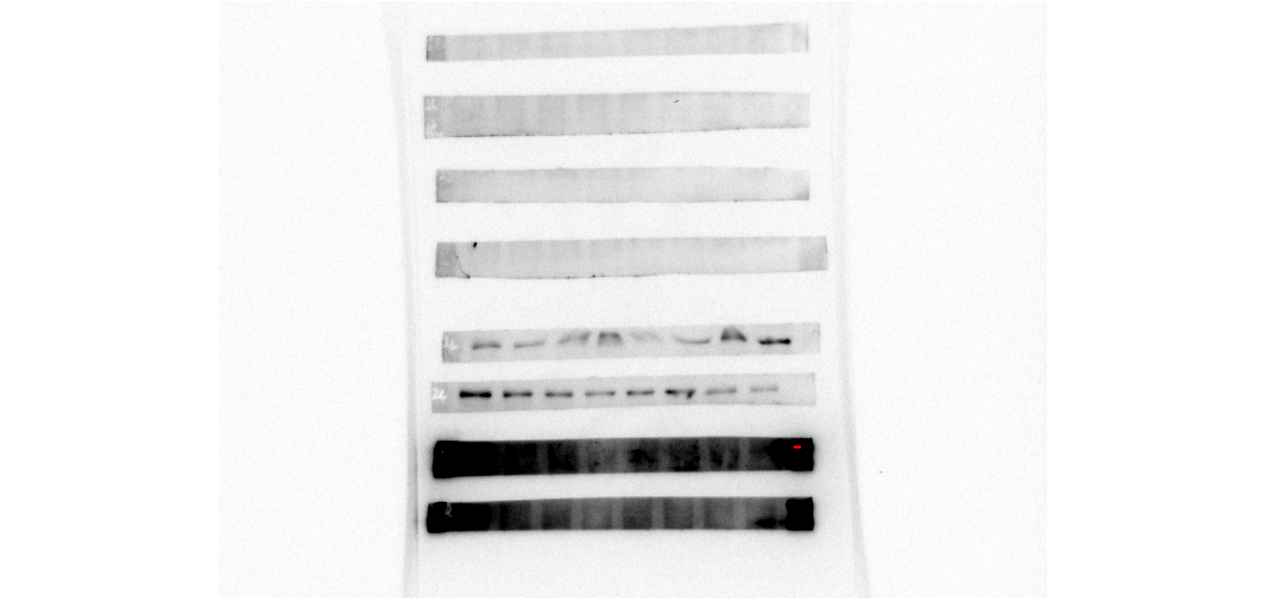


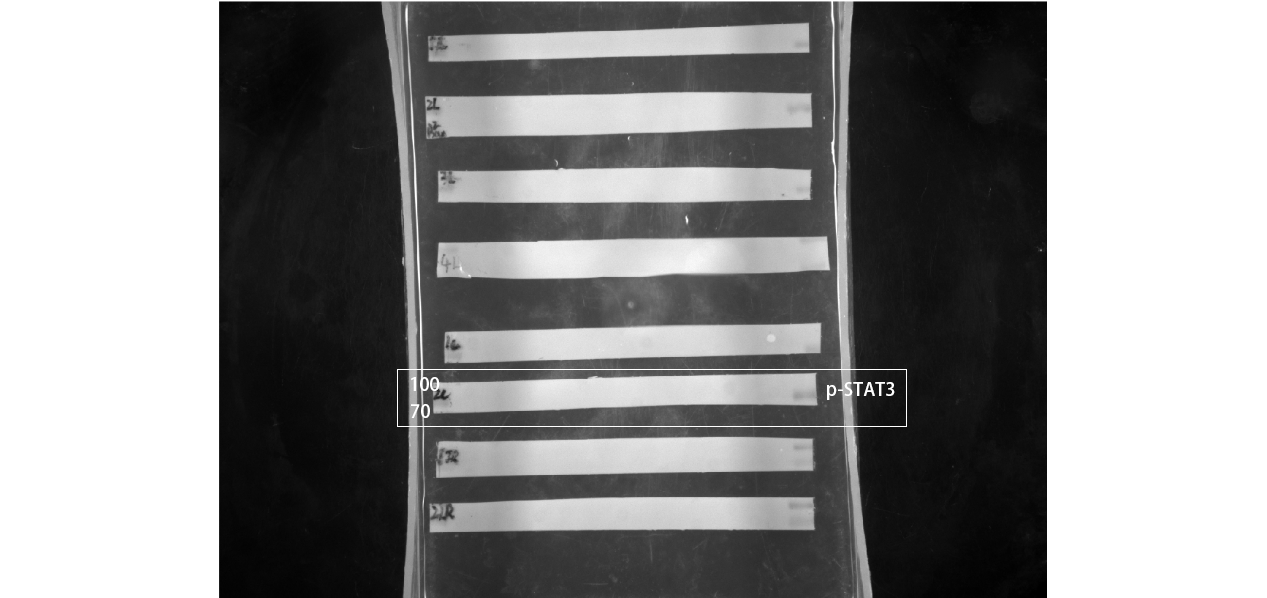


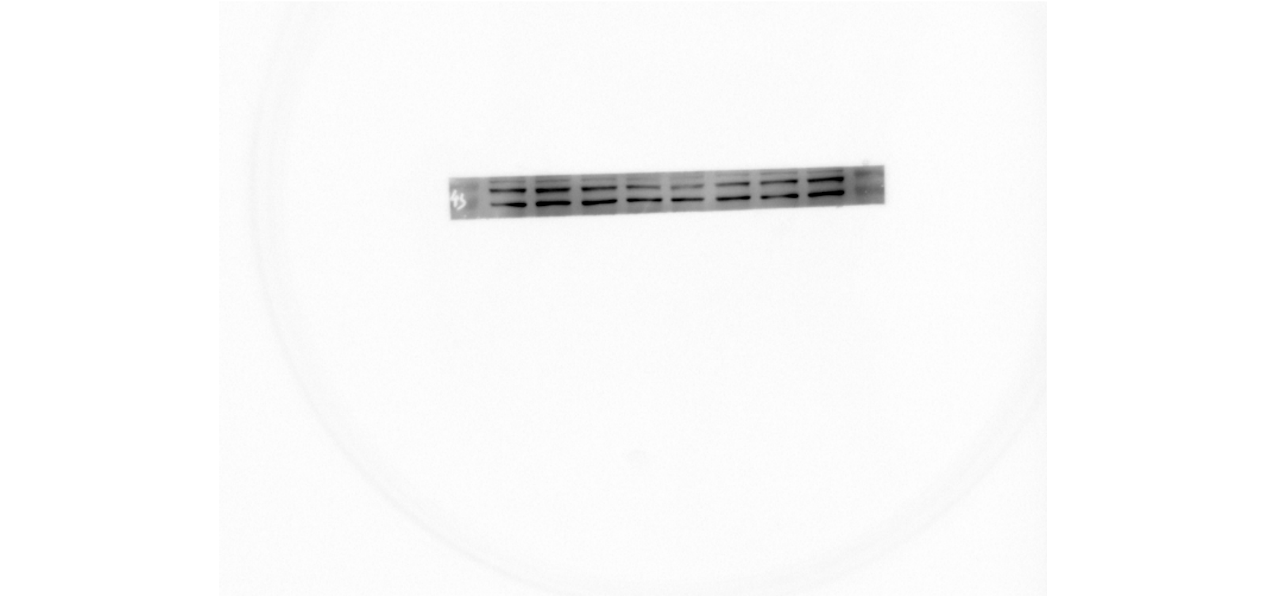


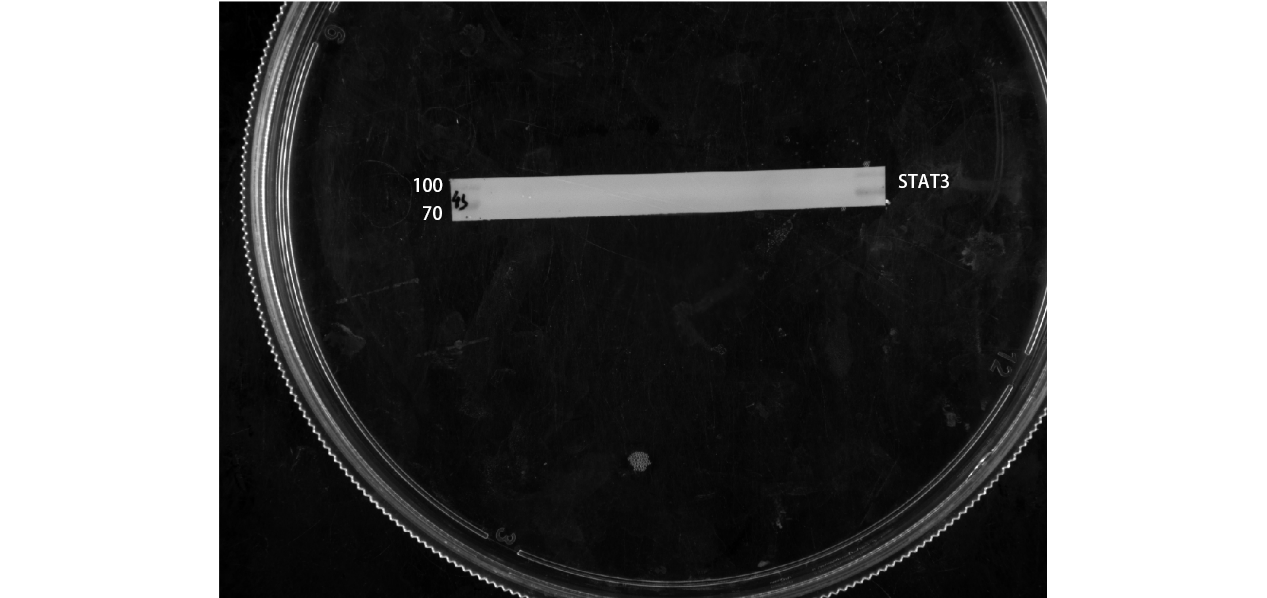


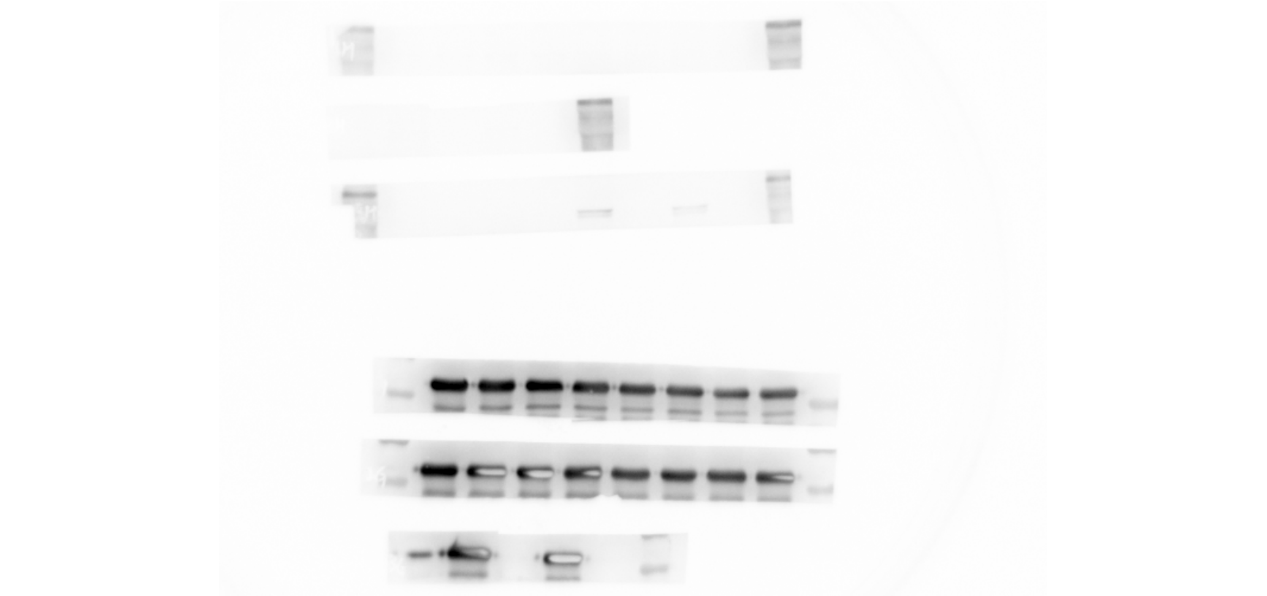


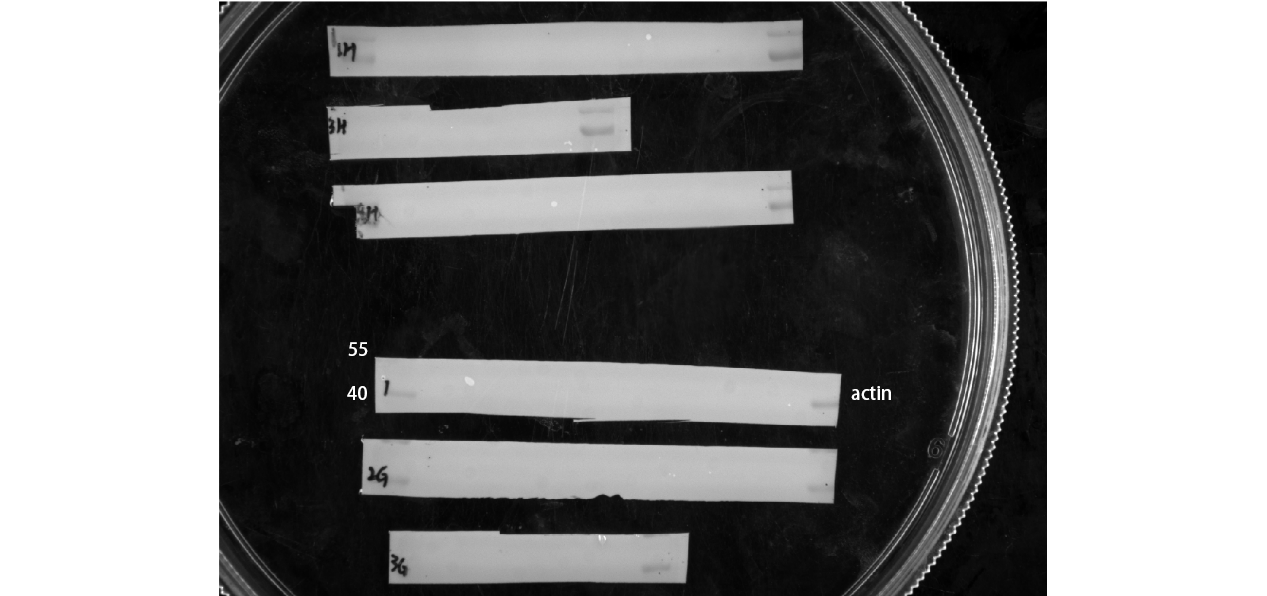


FigS1D


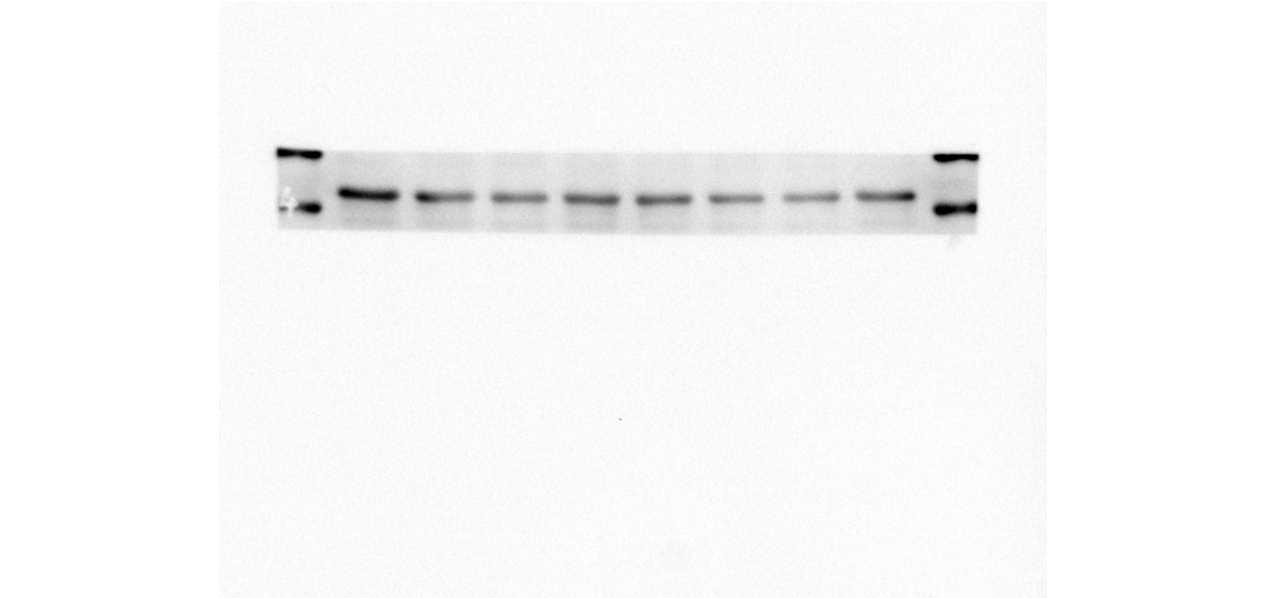


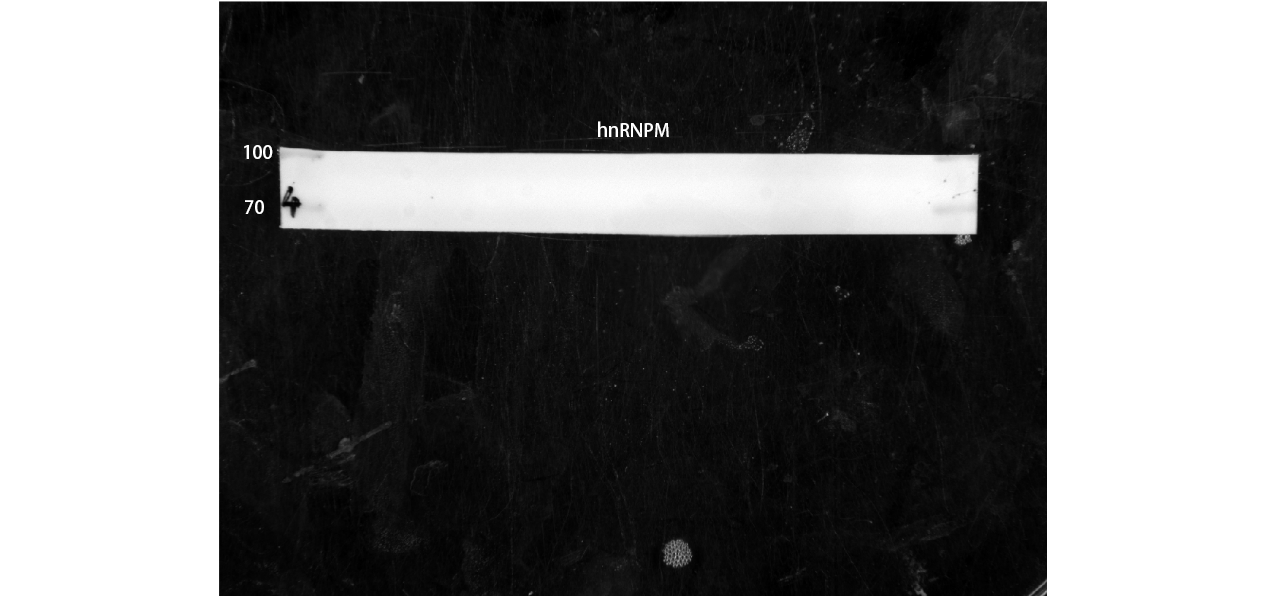


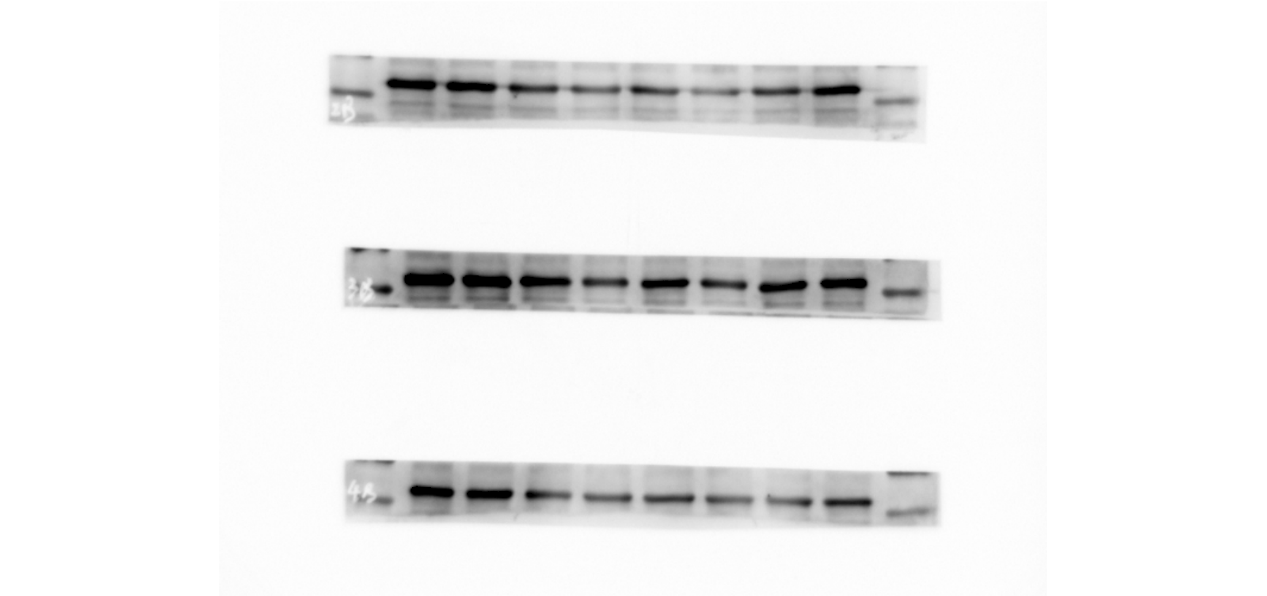


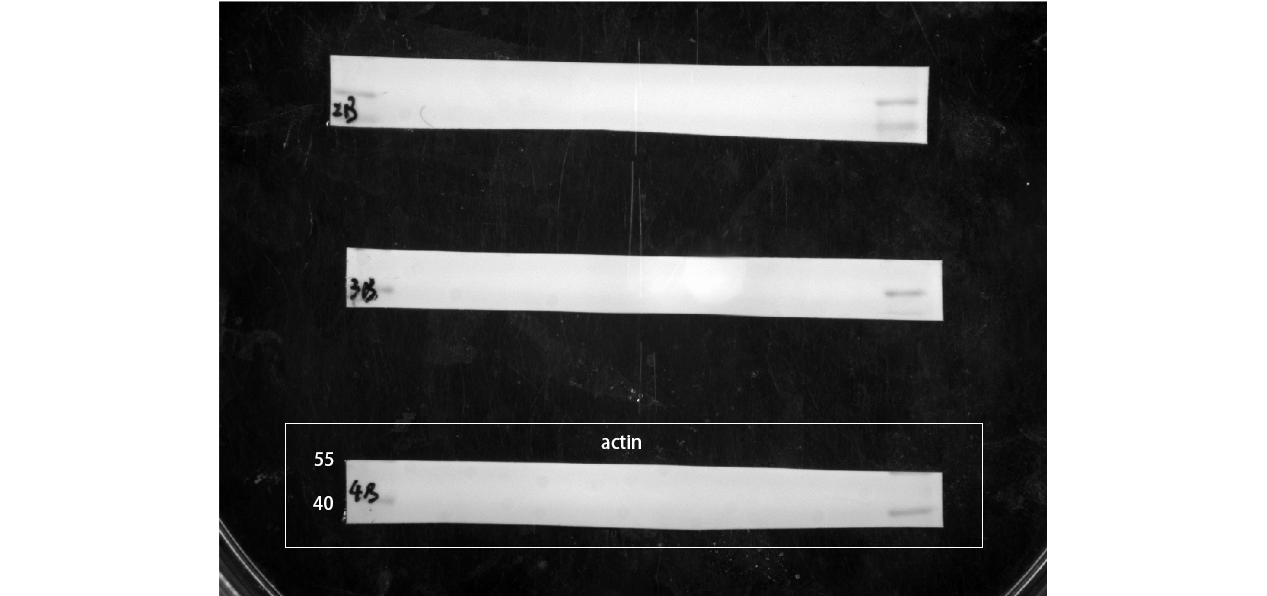


FigS1E


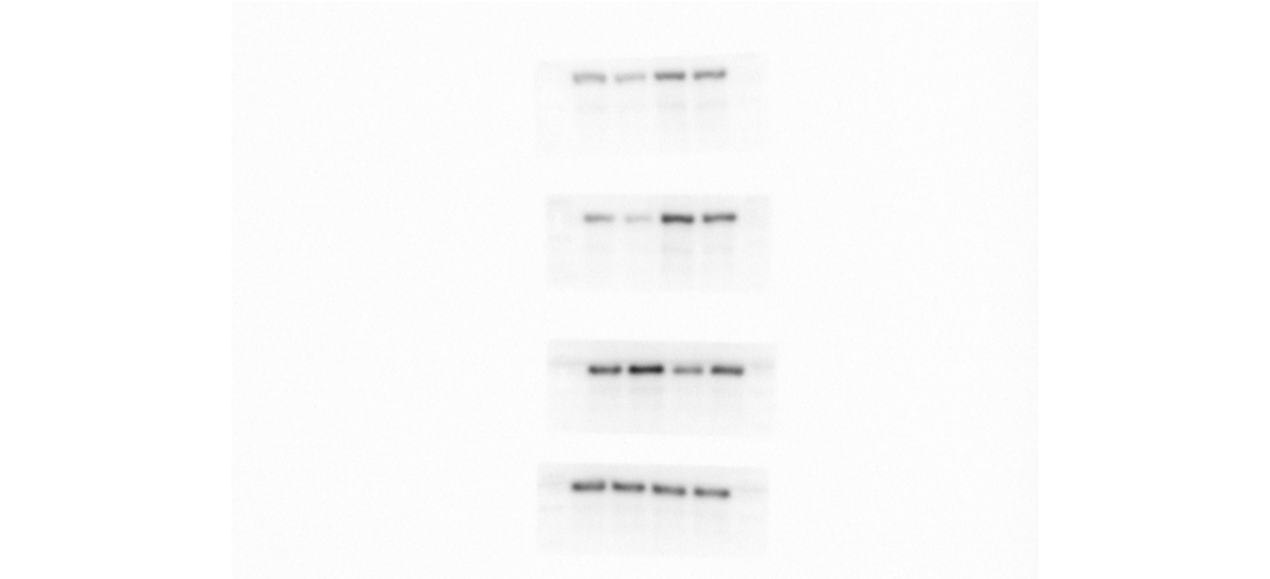

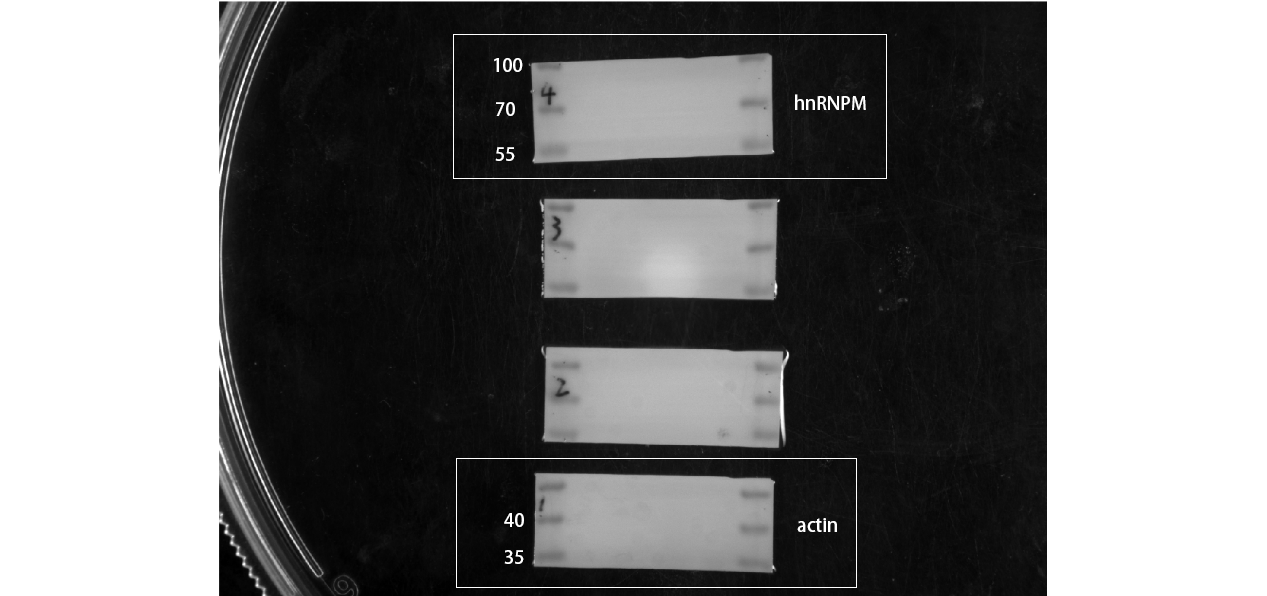

Supplement: Supplementary file 5 — Protein gel original data [file 41419_2023_5717_MOESM5_ESM.doc]
