# Supplementary material for: circPTPN12 promotes the progression and sunitinib resistance of renal cancer via hnRNPM/IL-6/STAT3 pathway
Source: Cell Death Dis. 2023 Mar 31;14(3):232. doi: 10.1038/s41419-023-05717-z (PMC10066201; doi:10.1038/s41419-023-05717-z)
Supplement: Supplementary file 2 — Supplementary Figure Legends [file 41419_2023_5717_MOESM2_ESM.doc]

**Supplementary Figure Legends**

**Fig. S1**

**a** The precipitates immunoprecipitated by anti-hnRNPM antibody confirmed the existence of hnRNPM in circPTPN12 pulldown products. **b, c** GSEA displayed the biological processes and signaling pathways of hnRNPM in KIRC. **d, e** circPTPN12 and hnRNPM did not affect each other's level of expression. **f** The change of three isoforms of IL-6 mRNA after knocking down hnRNPM.
