# Supplementary material for: circPTPN12 promotes the progression and sunitinib resistance of renal cancer via hnRNPM/IL-6/STAT3 pathway
Source: Cell Death Dis. 2023 Mar 31;14(3):232. doi: 10.1038/s41419-023-05717-z (PMC10066201; doi:10.1038/s41419-023-05717-z)
Supplement: Supplementary file 3 — Supplementary table1 to 5 [file 41419_2023_5717_MOESM3_ESM.doc]

**Supplementary tables**

Supplementary Table 1. siRNA sequences used in this study.

| Name | si RNA sequences (5’ - 3’) | Company |
| --- | --- | --- |
| si-circPTPN12-1 | GTATTCATTGCAGATCATT | RiboBio |
| si-circPTPN12-1 | CATTGCAGATCATTGTAAT | RiboBio |
| si-circPTPN12-1 | TGCAGATCATTGTAATGGC | RiboBio |
| si-hnRNPM-1 | CUGUGCAAGCUAUAUCUAUGUdTdT | QijingBio |
| si-hnRNPM-2 | CGAAUUGAUAGAAACGCUUAAdTdT | QijingBio |
| si-hnRNPM-3 | CCUUUGGUGGUGGUAUGGAAAdTdT | QijingBio |

Supplementary Table 2. Primers sequences used in this study.

| Name | Forward-primer (5’ - 3’) | Reverse-primer (5’ - 3’) |
| --- | --- | --- |
| GAPDH | CAATGACCCCTTCATTGACC | TTGATTTTGGAGGGATCTCG |
| Divergent GAPDH | TCAAGAAGGTGGTGAAGCAGGC | ATGCCAGTGAGCTTCCCGTT |
| circPTPN12 | GGCCAGACCATGATGTTCCTT | CTCAAATTCTCGGCAGGCCA |
| mPTPN12 | CTGACCACAATGGGGAGGAC | CGGCTGTGATCAAATGGCAG |
| lin-PTPN12 | CGTCTATGGGCCAAAAGCAT | CACCTGTTCTTCCACAGCCT |
| PTPN12 | AGTTGCCTTGTTGAAGGGGAT | AGAAGGTGTCAAGATGGGTGG |
| U6 | TGCGGGTGCTCGCTTCGGCAGC | CCAGTGCAGGGTCCGAGGT |
| IL6 | ACTCACCTCTTCAGAACGAATTG | CCATCTTTGGAAGGTTCAGGTTG |
| IL8 | TTTTGCCAAGGAGTGCTAAAGA | AACCCTCTGCACCCAGTTTTC |
| LIF | CCAACGTGACGGACTTCCC | TACACGACTATGCGGTACAGC |
| IL11 | CGAGCGGACCTACTGTCCTA | GCCCAGTCAAGTGTCAGGTG |
| CNTF | GAAGATTCGTTCAGACCTGACTG | AAGGTTCTCTTGGAGTCGCTC |
| OSM | CACAGACTGGCCGACTTAGAG | AGTCCTCGATGTTCAGCCCA |
| TLR4 | AGACCTGTCCCTGAACCCTAT | CGATGGACTTCTAAACCAGCCA |
| hnRNPM | GCGGCGACGGAGATCAAAA | CTCATTCTGAGCAGGTCGTTC |
| IL6 isoform 2 | ACTCCTTCTCCACAAACATGTAAC | GACCAGAAGAAGGAATGCCCA |
| IL6 isoform 3 | AACTCCTTCTCCACAATACCCC | ATCTGAGGTGCCCATGCTAC |

Supplementary Table 3. Antibodies used in this study.

| Name | Company | Catalog Number | Assay |
| --- | --- | --- | --- |
| STAT3 | Abclonal | A19566 | WB |
| p-STAT3 | Abclonal | AP0715 | WB, IHC |
| PARP, Cleaved PARP | Proteintech | 66520-1-Ig | WB |
| Caspase 3, Cleaved Caspase 3 | Abclonal | A11953 | WB |
| hnRNPM | Proteintech | 26897-1-AP | WB, RIP, IF |
| β-actin | Proteintech | 66009-1-Ig | WB |
| HRP-conjugated Affinipure Goat Anti-Mouse IgG(H+L) | Proteintech | SA00001-1 | WB |
| HRP-conjugated Affinipure Goat Anti-Rabbit IgG(H+L) | Proteintech | SA00001-2 | WB |
| ABflo^TM^ 488-conjugated Donkey Anti-Rabbit IgG (H+L) | Abclonal | AS035 | IF |
| Monoclonal ANTI-FLAG M2 antibody | Sigma-Aldrich | F3165 | RIP |

Supplementary Table 4. Biotin probes used in this study.

| Name | Sequence (5’ - 3’) | Company |
| --- | --- | --- |
| Sense probe | CGGCAGGCCATTACAATGATCTGCAATGAATACAAATAGG | Tsingke |
| Anti-sense probe | CCTATTTGTATTCATTGCAGATCATTGTAATGGCCTGCCG | Tsingke |

Supplementary Table 5. Plasmids and lentivirus used in this study.

| Name | Structure or sequence | Company |
| --- | --- | --- |
| Full length or truncated plasmids | pcDNA3.1-hnRNPM(ΔRRM)-AMP-3xFlag | genecreate |
| circPTPN12 over-express  lentivirus | CMV-circPTPN12-EF1a-ZsGreen1-T2A-puromycin | genechem |
